# RIPK2 promotes colorectal cancer metastasis by protecting YAP degradation from ITCH-mediated ubiquitination

**DOI:** 10.1038/s41419-025-07599-9

**Published:** 2025-04-04

**Authors:** Chen Lu, Hongda Liu, Tianyu Liu, Sizheng Sun, Yanan Zheng, Tao Ling, Xiagang Luo, Yiming E, Yuting Xu, Jie Li, Lei Liu, Lin Miao, Zhengxia Liu, Chunzhao Yu

**Affiliations:** 1 Department of General Surgery, Sir Run Run Hospital of Nanjing Medical University, Nanjing, 211112 Jiangsu China; 2 Department of General Surgery, The Second Affiliated Hospital of Nanjing Medical University, Nanjing, 210011 Jiangsu China; 3 Department of General Surgery, First Affiliated Hospital of Nanjing Medical University, Nanjing, 210029 Jiangsu China; 4 Department of General Surgery, The Affiliated Taizhou People’s Hospital of Nanjing Medical University, Taizhou, 225300 Jiangsu China; 5 Department of Geriatrics, The Second Affiliated Hospital of Nanjing Medical University, Nanjing, 210011 Jiangsu China; 6 Ophthalmic Oncology Department, Nanjing Medical University Eye Hospital, Nanjing, 210008 China; 7 Department of Oncology, The Second Affiliated Hospital of Nanjing Medical University, Nanjing, 210011 Jiangsu China; 8 Department of Gastroenterology, The Affiliated Yixing Hospital of Jiangsu University, Yixing, 214200 Jiangsu China; 9 Medical Centre for Digestive Diseases, The Second Affiliated Hospital of Nanjing Medical University, Nanjing, 210011 Jiangsu China

**Keywords:** Oncogenes, Ubiquitylation

## Abstract

Colorectal cancer (CRC) is the second leading cause of cancer-related death worldwide, making the exploration of metastatic mechanisms crucial for therapeutic advancements. In this study, we identified receptor-interacting protein kinase 2 (RIPK2) as an independent risk factor for poor CRC prognosis. Single-cell RNA sequencing and spatial transcriptomics revealed that a tumor cell cluster with high RIPK2 expression exhibited enhanced metastatic potential, closely linked to bacterial invasion. In vitro and in vivo experiments confirmed that RIPK2 specifically promotes tumor cell migration and invasion, rather than proliferation. Proteomic analysis indicated that RIPK2 knockdown leads to increased proteolysis mediated by ubiquitin, particularly affecting the oncoprotein YAP. Additionally, bacterial invasion of epithelial cells was significantly suppressed in RIPK2 knockdown cells, suggesting a connection to the NOD2-RIPK2 pathway, stimulated by bacterial muramyl dipeptide (MDP). We demonstrated that MDP levels are significantly higher in CRC tissues compared to adjacent non-cancerous tissues, correlating with RIPK2 activation. This activation triggers K63-linked ubiquitination of RIPK2, essential for NF-κB and MAPK pathway activation. Mechanistic studies identified the E3 ubiquitin ligase ITCH as a critical mediator, balancing K63-linked ubiquitination of RIPK2 and K48-linked ubiquitination of YAP, leading to YAP degradation and suppressed CRC metastasis. The stability of YAP could also be disrupted by GSK583, a pharmacological inhibitor of RIPK2, effectively suppressing CRC metastasis. Our findings provide deep insights into RIPK2’s role in CRC progression and present a promising target for future therapeutic strategies.

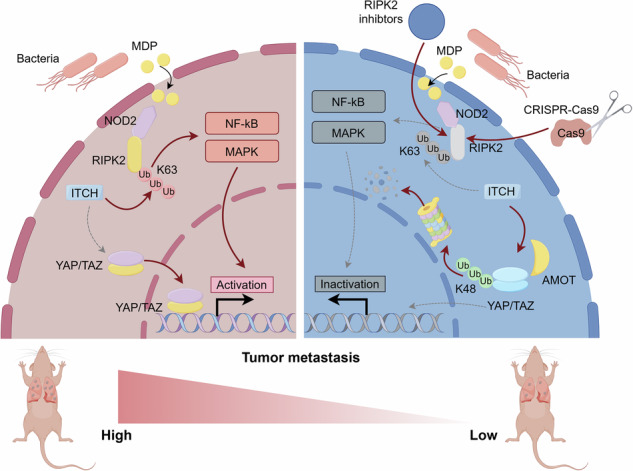

## Introduction

Colorectal cancer (CRC) is one of the common malignant tumors that threaten the health and life of humans and has high incidence and mortality rates among malignant tumors [1]. Although the overall survival (OS) rate of colorectal cancer has improved to some extent with the advancement of diagnostic and treatment technologies [2], nearly one-fourth of CRC patients have tumor metastasis at the time of initial diagnosis and around 50% undergo tumor metastasis during the treatment [3, 4]. Furthermore, the prognosis of metastatic colorectal cancer patients is very poor, with a 5-year survival rate of only 14% [5], which mainly depends on the molecular subtype of the tumor and the patient’s own health status [6]. Therefore, it is very urgent to find genes responsible for metastatic CRC (mCRC) so that targeted therapies can be applied for CRC treatment.

With the advent of robust next‑generation sequencing techniques and molecular biology techniques, numerous genes directly or indirectly linked with CRC have now been identified. Almost all these genes are protein kinases or associated with protein kinases [7–10]. This is also the case for the genes identified in other cancers. The reason that protein kinases account for a high proportion in the identified genes in cancers may be simply because protein kinases are rich genes (over 518 protein kinases presented in human, which constitute about 2% of all human genes) and especially they participate in the regulation of almost every aspect of cell life, such as proliferation, cell cycle, apoptosis, motility, growth, differentiation, and so on [11–13]. Any alternation in sequences, expressions or activities of kinases could be oncogenic or generate cancers [14]. For example, mutated kinase BRAF (V600E) resulted in colorectal cancer and other several cancers [15]. Thus, identification of more protein kinases in CRC will be very helpful and essential for diagnosis and targeted therapeutics for CRC treatment.

In the present study, we combined bioinformatics, clinical data, and various experiments to discover a receptor-interacting protein kinase, RIPK2, important especially for CRC metastasis. Previously, RIPK2 was shown to play an essential role in intestinal immunity and inflammation through mediating the typical signal transduction by nucleotide-binding oligomerization domain-containing proteins 1 and 2 (NOD1/2), which are activated by bacterial sensing pattern recognition receptors [16, 17], but not functions in CRC and other cancer types. Through single-cell RNA sequencing (scRNA-seq) and spatial transcriptomics analyses, we identified a subcluster of tumor cells characterized by high RIPK2 expression. This subcluster was positioned at the terminal stage of tumor cell differentiation and exhibits highly invasive and metastatic capabilities. Subsequent in vitro and in vivo experiments confirmed that RIPK2 is essential for colorectal cancer cell metastasis, but not for proliferation. Interestingly, both enrichment analysis of the RIPK2-characterized tumor cell cluster from scRNA-seq and proteomic analysis of HCT116 cells with RIPK2 knockdown revealed significant activation of pathways related to bacterial invasion of epithelial cells and Salmonella infection. This suggests that RIPK2 is crucial for mediating the impact of bacterial invasion on colorectal cancer metastasis. It is well known that RIPK2’s activity is closely associated with muramyl dipeptide (MDP) from bacteria cell wall components [18, 19]. Based on this, we discovered that MDP levels are significantly higher in colorectal cancer tissues compared to adjacent non-cancerous tissues, suggesting the potential presence of the classical NOD2-RIPK2 signaling pathway in CRC cells, and K63-linked ubiquitination of RIPK2 is essential for this process. Surprisingly, proteomic analysis indicated inhibiting RIPK2 not only blocks the MDP-activated signaling pathway, but promotes the degradation of the oncoprotein YAP via the proteasomal pathway. We found that the E3 ubiquitin ligase ITCH plays a crucial dual role in this process. Notably, ITCH is essential for K63-linked ubiquitination of RIPK2 [20], and also serves as the primary ubiquitin ligase responsible for K48-linked ubiquitination of YAP [21, 22]. Here, we found a novel mechanism with the fact of RIPK2 stabilizing YAP protein in CRC. In the presence of RIPK2, ITCH primarily facilitates K63-linked ubiquitination of RIPK2, leading to the activation of NF-κB and MAPK signaling pathways. However, when RIPK2 is inhibited, ITCH is redirected by AMOT to catalyze K48-linked ubiquitination of YAP, resulting in the suppression of CRC metastasis. Taken together, RIPK2 could be a promising therapeutic target in mCRC.

## Materials and methods

### Bioinformatics analysis

We screened some datasets with one-to-one matching samples from public resources, such as Gene Expression Omnibus (GEO) database, TCGA database and Clinical Proteomic Tumor Analysis Consortium (CPTAC) database, to conduct bioinformatics analysis, including bulk RNA-seq analysis, scRNA-seq analysis, and spatial transcriptomics analysis. All detailed bioinformatics analysis methods are described in Supplementary materials and methods.

### Collection of patient samples

A total of 158 CRC tissues and paired adjacent normal tissues were collected from CRC patients who underwent radical operation at the Second Affiliated Hospital of Nanjing Medical University between January 2015 and December 2020. However, 6 patients were excluded, including 2 patients with less than 12 lymph nodes removed and 4 patients who did not receive the standardized postoperative treatment. The clinical information is provided in the Additional Supplementary Files 1. All samples were divided into two sections, which one part was preserved in liquid nitrogen for RNA and protein extraction, and the other part was fixed with formalin and sectioned for staining. Acquisition of all samples was approved by the Ethics Committee of the Second Affiliated Hospital of Nanjing Medical University (No.2020KY092), and received informed consent from each patient.

### Cell lines and cell culture

HCT116 (#FH0027), SW480 (#FH0022), SW620 (#FH0021), DLD-1 (#FH0017) cells were obtained from FuHeng Biotechnology Co., LTD (Shanghai, China). NCM-460, HT-29, Lovo and CaCO2 cells were presented by the Lab center of the Second Affiliated Hospital of Nanjing Medical University. All cell lines have been identified by Short Tandem Repeat (STR) analysis. The cell lines we purchased were cultured according to the instruction manual.

### RNA isolation and real-time quantitative PCR (RT-qPCR)

Total RNA from tissues or cells was extracted using Trizol (#15596026CN, Invitrogen, USA). The cDNA was synthesized by reverse transcription of total RNA using 5×HiScript II qRT SuperMix (#R222-01, Vazyme). Subsequently, RT-qPCR was performed using SYBR qPCR Master Mix (#Q421-02, Vazyme) in a LightCycler 480 Real-time PCR Detection Systerm (Roche, Switzerland). The relative gene expression was normalized to GAPDH using the 2-^ΔΔCT^ method. Gene-specific primers are provided in Supplementary materials and methods.

### Western blot

Tissues or cells were lysed to extract their protein. The protein samples were separated by SDS-PAGE gel System (Bio-Rad) and transferred to 0.45 μm polyvinylidene difluoride membranes (Millipore). Then, the membranes were blocked with 5% bovine serum albumin (BSA) for around 2 h at room temperature, followed by probing the membranes overnight at 4 °C with primary antibodies. Next, the membranes were incubated with secondary antibodies for an hour at room temperature, and then were washed with Tris-buffered saline-tween 20 (TBST) three times for half an hour. The signal was visualized with ECL Chemiluminescence Kit (#E423-01, Vazyme). All antibodies are described in Supplementary materials and methods.

### Transient transfection and lentiviral infection

Lipofectamine 3000 (Invitrogen, Carlsbad, USA) was used to transfect small inference RNAs (siRNAs) or plasmids into cells transiently, when cells grew to a confluency of 50%-70%. For lentiviral infection, the lentivirus was diluted in the medium with fetal bovine serum to the desired concentration (HCT116, MOI = 5 ~ 10; SW480, MOI = 20 ~ 50), and then, polybrene (Final concentration was 2 ~ 10 μg/ml) (Beyotime) was added in it to enhance efficiency of infection. All siRNAs, plasmids and lentivirus constructed or purchased are detailed in Supplementary materials and methods.

### Cell proliferation assay

Cell Counting Kit-8 (CCK-8) (#A311-01, Vazyme) was used to perform cell proliferation assay, according to the manufacturer’s instructions. Clonogenic assay was also used to detect cell proliferation. When cells were successfully infected with lentivirus, 2 ml of them (1000/ml) were seeded in 6-well plates and cultured in 10% FBS-containing media and 1% penicillin-streptomycin. The media must be replaced each 2 days, and then, cells were fixed in 4% paraformaldehyde for 15 min, followed by staining cells with crystal violet solution (#C0121, Beyotime) for 15 min.

### Transwell assay

Cell migration and invasion assays were performed using 6.5 mm Transwell with 8.0 μm pore polycarbonate membrane insert (#CLS3422, Corning). For migration assay, we added 750 μL 10% FBS-containing medium to the lower chamber, followed by seeding 200 μL cell suspension (8 × 10^4^/ml) without FBS into the upper chamber. Subsequently, incubating them at 37 °C for 48 h, these cells were fixed in 4% paraformaldehyde and then stained with crystal violet solution. Finally, the stained cells in the upper chamber were gently wiped away, and the stained cells on the outside membrane of chambers were photographed and counted under an optical microscope. For invasion assay, before seeding cells into the upper chamber, we used 50 μL Matrigel matrix (diluted at 1:10) (#356234, Corning) to coat the inside membrane of the chamber. The remaining steps are similar to cell migration assay.

### Animal studies

All animal experiments were approved by Institutional Animal Care and Use Committee (IACUC) at Nanjing medical university (No. IACUC-2404096), and their procedures were conducted in accordance with the guidelines of the Animal Care and Use Committee Guidelines. 6-week-old male BALB/c nude mice were chosen for our animal studies, including subcutaneous xenograft model and metastatic model. The detailed methods are described in Supplementary materials and methods.

### Hematoxylin and eosin (H&E) staining and immunohistochemistry (IHC)

The fixed tissues were embedded in paraffin, and sectioned at 4 μm thickness. For H&E staining, sections were deparaffinized, rehydrated, stained with hematoxylin for 5 min, and then with eosin for 2 min. For IHC assay, sections underwent antigen retrieval using citrate buffer (pH 6.0) at 95 °C for 20 min. Endogenous peroxidase was blocked with 3% hydrogen peroxide, and nonspecific binding was prevented using 5% BSA. The slides were incubated by primary antibodies and secondary antibodies, then stained by 3,3′-diaminobenzidine (DAB). The stained slides were digitally scanned using NanoZoomer®S360 (HAMAMATSU PHOTONICS) for observation and analysis. IHC staining was evaluated by calculating the percentage of positively stained tumor cells (proportion of scores (PS)) and the staining intensity (intensity scores (InS)), scoring each sample with the formula of PS×InS [23]. Two pathologists, blinded to clinical and grouping data, conducted both pathological diagnoses and IHC scoring to minimize observer bias. The antibodies are described in Supplementary materials and methods.

### Statistical analysis

R (v4.2.2), GraphPad Prism (v8.0) and SPSS (v23.0) were applied to perform statistical analysis. Continuous variables were expressed as mean ± standard deviation (SD) and compared using the student’s *t* test. Categorical variables were expressed as frequencies, and comparisons were made using the Chi-square test or Fisher’s exact test. All statistical tests were two-tailed and *p* value < 0.05 was considered statistically significant.

A complete description of the materials and methods, including bioinformatics analysis, gene-specific primers, antibodies, animal studies, Data-independent acquisition (DIA) proteomics, co-immunoprecipitation (Co-IP), k48-ubiquitination assay, MDP Concentration measurement, immunofluorescence (IF), and Kaplan-Meier survival analysis, is available in Supplementary materials and methods.

## Results

### Screening for differentially expressed protein kinases in CRC samples

**Fig. 1:**
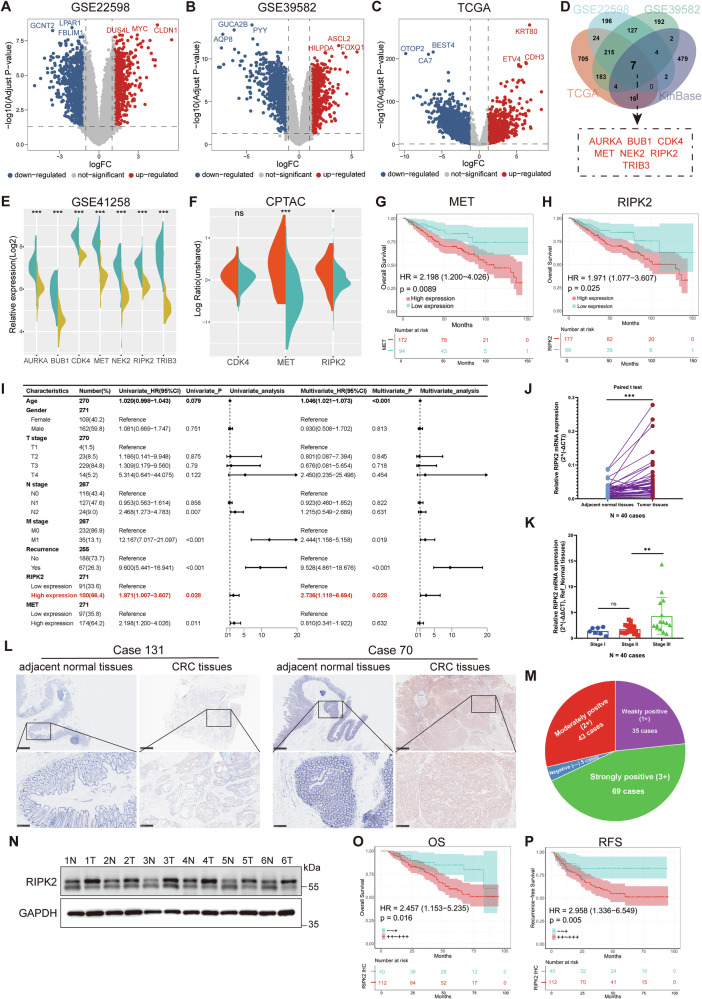
RIPK2 is identified as an independent risk factor for poor prognosis of CRC. **A**–**C** Volcano plot of DEGs identified from GSE22598, GSE39582 and TCGA (COAD and READ) datasets. Red pots express upregulated DEGs, and blue pots express downregulated DEGs under the same thresholds. The gray dots denote genes that are not differentially expressed. |log2FC | > 1.0 and adjusted *P* value < 0.05 were set as the cut-off criteria. **D** The intersection of differentially protein kinase genes of GSE22598, GSE39582, TCGA (COAD and READ) datasets and KinBase database. **E** Seven differentially expressed protein kinase genes (AURKA, BUB1, CDK4, MET, NEK2, RIPK2, TRIB3) at mRNA levels were examined between tumor and paired adjacent normal tissues using the GSE41258 dataset. **F** Four of seven genes (AURKA, BUB1, NEK2, TRIB3) were not existed in CPTAC database (PDC000116), the other genes (CDK4, MET, RIPK2) at protein levels were assessed between tumor and paired adjacent normal tissues. Overall survival analysis of MET (**G**) and RIPK2 (**H**) in CRC cohorts, including GSE39582, GSE41258, GSE87211 and TCGA (COAD and READ), using Kaplan-Meier survival analysis. **I** Univariate and multivariate Cox proportional regression analyses for OS in CRC cohorts was visualized by forest plot. **J** RIPK2 mRNA expression levels in 40 CRC primary tumor tissues and paired normal tissues were detected using RT-qPCR. **K** Expression analysis grouped by tumor stage of RIPK2 in 40 CRC cases were performed using RT-qPCR. **L** Representative images of IHC slides showed RIPK2 protein levels in CRC tumor tissues and normal tissues. **M** Pie chart figure showed the distribution of cases with RIPK2 protein levels, containing negative (–), weakly positive (+), moderately positive (++), and strongly positive (+++). **N** RIPK2 protein expression levels in 6 CRC primary tumor tissues and paired normal tissues were detected using western blotting. Kaplan-Meier survival analysis estimated the relationship between RIPK2 protein levels and OS (**O**) or RFS (**P**) in 152 CRC cases. *<0.05, **<0.01, ***<0.001, ****<0.0001.

Four colorectal cancer datasets (GSE22598, GSE39582, TCGA-COAD and TCGA-READ) were collected. All these datasets contained primary tumor lesions and paired adjacent normal samples. Analysis of common upregulated genes among these datasets (Fig. 1A–C) and then comparing them with the KinBase database identified seven differentially expressed protein kinase genes (Fig. 1D). Structural analysis of these differentially expressed genes showed AURKA, BUB1, CDK4, MET, NEK2, RIPK2, and TRIB3.

Next, the identified differentially expressed protein kinases were examined for their mRNA levels using GSE41258 dataset (Fig. 1E) and to determine their association with poor prognosis using Kaplan-Meier survival analyses. Two kinases (MET and RIPK2) with high mRNA abundance were significantly related to worse OS of CRC patients (Fig. 1G, H and Supplementary Fig. 1). We also examined their protein levels using CTPAC database, and two kinases (MET and RIPK2) showed significant differences (Fig. 1F).

### High expression of RIPK2 is an independent risk factor for poor prognosis of CRC patients

To determine whether RIPK2 or MET independently affects prognosis of CRC patients, we incorporated clinical data from five cohorts (GSE39582, GSE41258, GSE87211, TCGA-COAD, TCGA-READ) to perform univariate and multivariate Cox proportional regression analyses. High mRNA abundance of RIPK2 was identified as an independent risk factor for OS (Fig. 1I). Correlation between RIPK2 mRNA expression and clinical characteristics of five CRC cohorts was thus analyzed. The results showed that two factors (T stage and N stage) were significantly related to RIPK2 mRNA expression (Table 1). RT-qPCR analysis of RNA from 40 paired samples confirmed the higher RIPK2 mRNA levels in tumors, especially in Stage III samples (Fig. 1J, K).

**Table 1:** The correlation between relative RIPK2 mRNA expression and clinical characteristics of CRC in public resources (GSE39582 & GSE41258 & GSE87211 & TCGA).

Characteristics	Relative RIPK2 mRNA expression (Ref_paired normal tissues)		*P* value
	High expression (*N* = 180)	Low expression (*N* = 91)	
Age	64.131 ± 13.959	64.546 ± 9.814	0.777
Gender			0.362
Male	104	58	
Female	76	33	
T stage			0.009*
T1 & T2	24	3	
T3 & T4	156	87	
N stage			<0.001*
N0	82	34	
N1	73	54	
N2	24	0	
M stage			0.342
M0	152	80	
M1	26	9	
TNM stage			0.186
I & II	76	30	
III & IV	102	58	
Recurrence			0.653
NO	122	66	
YES	46	21	

Next, IHC analysis of RIPK2 protein expression was performed in 152 CRC cases. As shown by IHC, the levels of RIPK2 were higher in CRC tissues than in adjacent normal tissues (Fig. 1L). In addition, 5 of these cases were negative (–), while 35 were weakly positive (+), 43 moderately positive (++), and 67 strongly positive (+++) (Fig. 1M). Moreover, protein samples from 6 patients (2 Stage II, 4 Stage III) revealed RIPK2 expression was higher in tumor tissues than in their adjacent normal tissues (Fig. 1N). Similar to clinical findings of RIPK2 mRNA expression, high protein abundance of RIPK2 was associated with worse OS and RFS of CRC patients (Fig. 1O, P), and clinical characteristics of N stage, TNM stage, and recurrence (Table 2). It was identified as an independent risk factor for RFS in 152 CRC patients (Tables 3, 4). Thus, RIPK2 overexpression can serve as an independent risk factor for poor prognosis in CRC.

**Table 2:** Correlation between RIPK2 protein expression level and clinical characteristics of CRC.

Characteristics	RIPK2 protein expression level		*P* value
	─ ~ + (*N* = 40)	++ ~ +++ (*N* = 112)	
Age	61.725 ± 10.276	63.670 ± 10.056	0.305
Gender			0.798
Male	23	67	
Female	17	45	
T stage			0.663
T1 & T2	3	11	
T3 & T4	37	101	
N stage			0.021*
N0	24	40	
N1	10	53	
N2	6	19	
TNM stage			0.027*
I	3	6	
II	21	34	
III	16	72	
Recurrence			0.004*
NO	33	64	
YES	7	48	
Vascular invasion			0.131
NO	39	98	
YES	1	14	
Perineural invasion			0.095
NO	33	77	
YES	7	35	
Histological differentiation			0.536
Well differentiated	2	10	
Moderately differentiated	25	74	
Poorly differentiated	13	28	
Tumor diameter/cm	5.225 ± 2.408	4.473 ± 1.837	0.078
Tumor site			0.858
Right-side	16	49	
Left-side	15	42	
Rectum	9	21

**Table 3:** Univariate and multivariate COX regression analysis of risk factors influencing RFS in CRC.

Characteristics	Number (%)	Univariate analysis		Multivariate analysis	
		HR (95%CI)	*P* value	HR (95%CI)	*P* value
Age	152	1.011 (0.984–1.039)	0.411	1.012 (0.984–1.042)	0.399
Sex					
Female	62 (40.8)	Reference		Reference	
Male	90 (59.2)	0.853 (0.502–1.451)	0.559	0.773 (0.436–1.370)	0.377
T stage					
T1 & T2	14 (9.2)	Reference		Reference	
T3 & T4	138 (90.8)	3.408 (0.830–13.998)	0.089	3.781 (0.893–16.012)	0.071
N stage					
N0	64 (42.1)	Reference		Reference	
N1	63 (41.4)	2.032 (1.075–3.840)	0.029	1.290 (0.645–2.582)	0.471
N2	25 (16.5)	2.707 (1.306–5.613)	0.007	1.517 (0.667–3.452)	0.32
Vascular invasion					
No	137 (90.1)	Reference		Reference	
Yes	15 (9.9)	2.684 (1.304–5.524)	0.007	1.598 (0.715–3.568)	0.253
Perineural invasion					
No	110 (72.4)	Reference		Reference	
Yes	42 (27.6)	2.601 (1.523–4.443)	<0.001	2.296 (1.289–4.088)	0.005
Histological differentiation					
Well differentiation	12 (7.9)	Reference		Reference	
Moderate differentiation	99 (65.1)	1.607 (0.495–5.214)	0.429	1.565 (0.468–5.228)	0.467
Poor differentiation	41 (27.0)	1.726 (0.500–5.963)	0.388	1.305 (0.356–4.786)	0.688
Tumor diameter	152	1.059 (0.935–1.201)	0.366	1.112 (0.955–1.296)	0.171
RIPK2 IHC grades					
“-” & “+”	40 (26.3)	Reference		Reference	
“++” & “+++”	112 (73.7)	2.945 (1.330–6.520)	0.008	2.665 (1.152–6.165)	0.022

**Table 4:** Univariate and multivariate COX regression analysis of risk factors influencing OS in CRC.

Characteristics	Number (%)	Univariate analysis		Multivariate analysis	
		HR (95%CI)	*P* value	HR (95%CI)	*P* value
Age	152	1.039 (1.010–1.070)	0.009	1.036 (1.004–1.068)	0.025
Sex					
Female	62 (40.8)	Reference		Reference	
Male	90 (59.2)	0.917 (0.527–1.596)	0.758	0.738 (0.403–1.351)	0.325
T stage					
T1 & T2	14 (9.2)	Reference		Reference	
T3 & T4	138 (90.8)	3.535 (0.857–14.575)	0.081	4.041 (0.956–17.080)	0.058
N stage					
N0	64 (42.1)	Reference		Reference	
N1	63 (41.4)	2.261 (1.184–4.315)	0.013	1.468 (0.716–3.010)	0.294
N2	25 (16.5)	2.031 (0.901–4.577)	0.087	1.235 (0.500–3.046)	0.647
Vascular invasion					
No	137 (90.1)	Reference		Reference	
Yes	15 (9.9)	1.878 (0.881–4.004)	0.103	1.311 (0.577–2.981)	0.517
Perineural invasion					
No	110 (72.4)	Reference		Reference	
Yes	42 (27.6)	2.311 (1.309–4.081)	0.004	2.225 (1.185–4.180)	0.013
Histological differentiation					
Well differentiation	12 (7.9)	Reference		Reference	
Moderate differentiation	99 (65.1)	0.608 (0.254–1.455)	0.264	0.676 (0.260–1.763)	0.424
Poor differentiation	41 (27.0)	0.637 (0.239–1.699)	0.367	0.612 (0.201–1.860)	0.386
Tumor diameter	152	1.044 (0.914–1.192)	0.523	1.108 (0.947–1.296)	0.201
RIPK2 IHC grades					
“–” & “+”	40 (26.3)	Reference		Reference	
“++” & “+++”	112 (73.7)	2.452 (1.151–5.225)	0.02	1.903 (0.842–4.303)	0.122

### Identification of the tumor epithelial cell cluster characterized by RIPK2 high expression

**Fig. 2:**
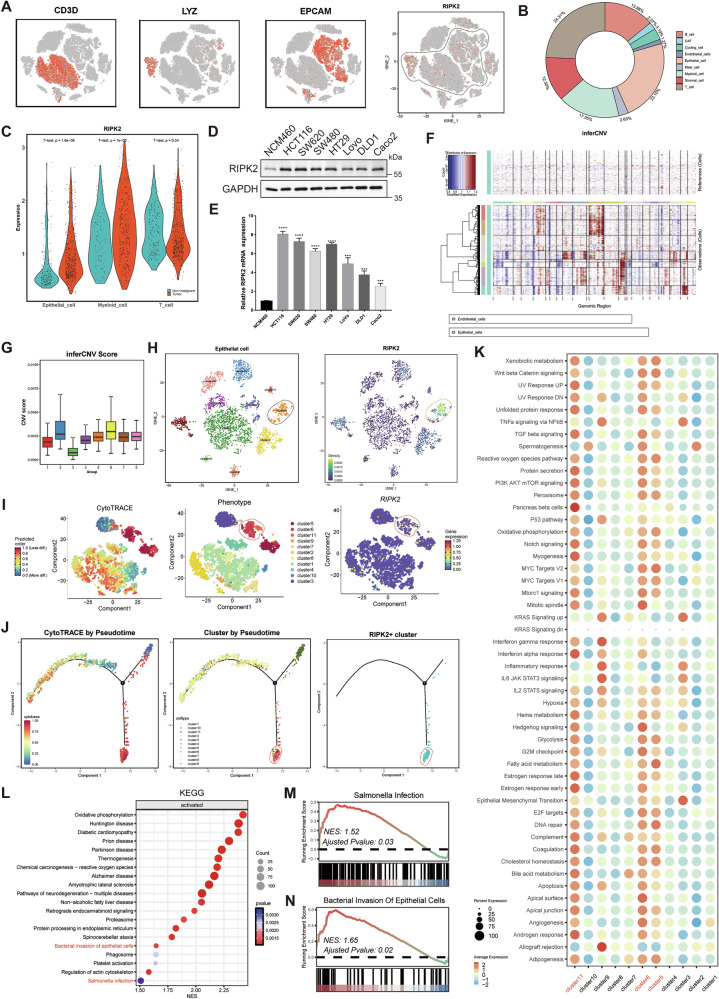
Identification of RIPK2^+^ subcluster in tumor epithelial cell clusters. **A** Expression of marker genes for the cell types (T cell, Myeloid cell, and Epithelial cell) defined above each panel, and t-SNE plots of whole cells, color-coded for the expression of RIPK2. **B** Pie chart figure showed the distribution of RIPK2 expression in 9 cell subclusters. **C** RIPK2 expression was assessed between tumor samples and non-malignant samples in 3 cell subclusters, containing T cell cluster, myeloid cell cluster, and epithelial cell cluster. **D** RIPK2 protein expression was detected in normal colonic epithelial cell line NCM460 and various CRC cell lines using western blotting. **E** RIPK2 mRNA expression was detected in normal colonic epithelial cell line NCM460 and CRC cell lines. **F** Heatmap showing large-scale CNV profile of each epithelial cluster and reference cell subcluster; the red and blue colors represent high and low CNV levels, respectively. **G** Box plot of CNV score of each group from tumor epithelial cell clusters. **H** t-SNE plot of cells with high CNV levels profiled here, with each cell color-coded for (left to right): its cell type of epithelial cells, and RIPK2 counts detected in these cells. **I** Differentiation trajectories obtained by CytoTRACE (left to right): visualization of CytoTRACE scores of malignant cells, distribution of 11 malignant cell subclusters, RIPK2 expression of malignant cells. Blue-green indicates lower scores (or lower expression) while red-yellow indicates higher scores (or higher expression). **J** Combined application of CytoTRACE and Monocle 2 to malignant cell differentiation (first panel), differentiation of all malignant cell clusters (second panel), and differentiation of the cell cluster with RIPK2 enrichment (third panel). **K** GSVA analysis of each malignant cell cluster. **L** KEGG enrichment analysis of differential expressed genes between RIPK2^+^ tumor cell cluster and cluster 3 by GSEA. **M**, **N** GSEA plots showing the activation of bacterial invasion of epithelial cells and salmonella infection in RIPK2^+^ subcluster, compared with cluster 3. *<0.05, **<0.01, ***<0.001, ****<0.0001.

In previously reported studies, the role of RIPK2 in colorectal cancer driven by inflammatory bowel disease (IBD) has been debated [24, 25], potentially due to its varying effects across different cell types. However, in the above clinical data analysis, we have identified high abundance of RIPK2 as an independent risk factor for poor prognosis in CRC, indicating that RIPK2 largely promotes CRC progression. Nevertheless, it is crucial to first determine in which specific cell types RIPK2 is differentially expressed and functionally contributes to the advancement of CRC. To address this, analysis of RIPK2 expression by scRNA-seq in 9 distinct cell types of each of six paired samples of advanced CRC showed that RIPK2 was primarily expressed in T cells (24.31%), epithelial cells (23.10%) and myeloid cells (17.05%) (Fig. 2A, B and Supplementary Fig. 2), but more different in epithelial and myeloid cells than in T cells between cancerous and normal tissues (Fig. 2C). Then we identified M1 macrophages, M2 macrophages, and dendritic cell subclusters within the myeloid cell cluster, but RIPK2 did not exhibit significant differential expression in any of these subclusters (Supplementary Fig. 3). In addition, RIPK2 was found to be predominantly expressed in Macrophages M1, known for their anti-tumor activity (Supplementary Fig. 3C). However, this finding did not align with the previous hypothesis that RIPK2 promotes CRC progression. Consequently, we directed our focus towards the tumor epithelial cell cluster.

To determine whether RIPK2 exhibits characteristic expression within the tumor epithelial cell cluster, we firstly verified RIPK2 mRNA and protein levels in CRC cell lines. Consistently, RIPK2 was overexpressed in CRC cell lines, particularly in highly metastatic cell lines, HCT116 and SW620 (Fig. 2D, E). Then, the inferCNV analysis identified tumor cell subclusters with notable copy number variations, followed by the identification of 11 malignant epithelial cell subclusters with dimensionality reduction and clustering (Fig. 2F, G). It is worth noting that RIPK2 was predominantly expressed in the cluster 6, a characteristic of this subcluster (Fig. 2H). Collectively, the differential expression of RIPK2 primarily originates from the tumor epithelial cell cluster, and a distinct subcluster of tumor epithelial cells characterized by RIPK2 can be identified.

### RIPK2^+^ tumor cell cluster possesses highly invasive and metastatic capabilities

To investigate what functional roles the RIPK2^+^ tumor cell cluster may have, we further analyzed all tumor cell subclusters by the cytoTRACE and pseudotime (Fig. 2I and Supplementary Fig. 4). The RIPK2+ cluster (cluster 6) is at the terminal end of the differentiation trajectory (Fig. 2I, J), probably developing from cluster 3 with less mutated. Then, GSVA analysis of this subcluster uncovered significant activation of various classical cancer signaling pathways (Fig. 2K). GSEA analysis on the differentially expressed genes between RIPK2^+^ cluster and cluster 3 revealed that bacteria invasion of epithelial cells and salmonella infection were significantly activated (Fig. 2L–N), which was consistent with previous studies reporting on bacterial activation of RIPK2 [26–28]. In summary, RIPK2^+^ cluster likely represents highly malignant tumor cells with significant invasive and metastatic capabilities.

**Fig. 3:**
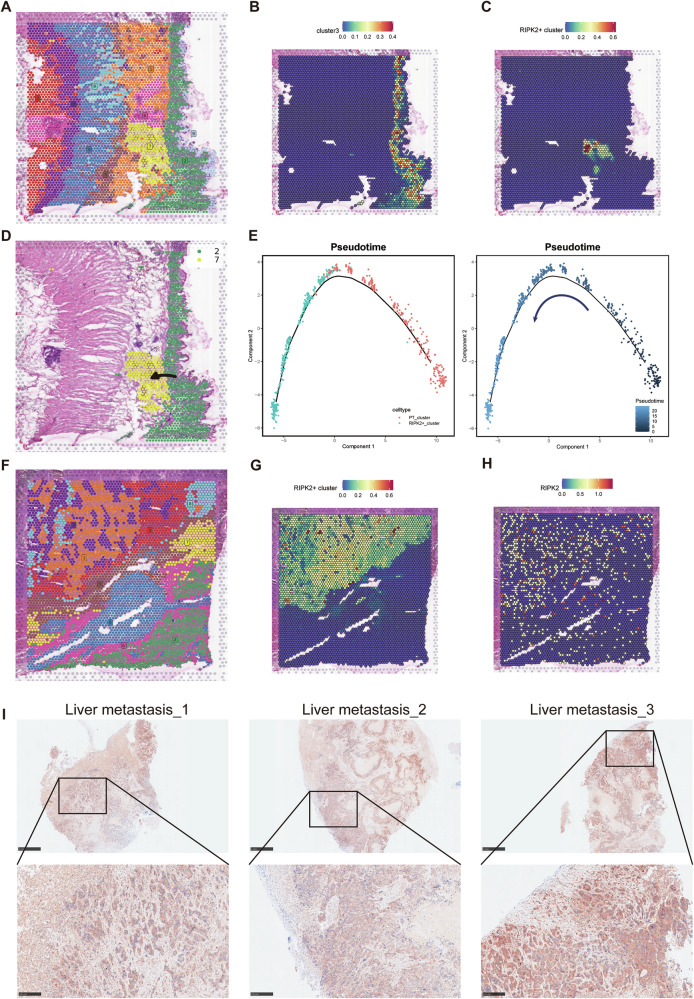
ST mapping and spatial trajectory analysis of RIPK2^+^ tumor cell cluster. The distribution map of clusters was generated using Louvain clustering and spot feature analysis methods in the primary lesion (**A**) and liver metastasis lesion (**F**). The spatial expression distribution map was generated using SPOTlight to predict the possible spatial distribution characteristics of the RIPK2^+^ tumor cell cluster (**C**) and the low-malignancy tumor epithelial cell cluster, cluster 3 (**B**). **D** Potential spatial differentiation trajectory from cluster 3 to the RIPK2^+^ tumor cell cluster. **E** Trajectory analysis of the RIPK2^+^ cluster over pseudotime, with different cell types distinguished by color (Left) and pseudotime indicated by color intensity (Right). The spatial distribution characteristics of RIPK2^+^ tumor cell cluster (**G**) and RIPK2 expression (**H**) were predicted using SPOTlight in the liver metastasis lesion. **I** Representative IHC images of RIPK2 in liver metastasis samples.

To determine whether RIPK2^+^ cluster exhibits invasive and metastatic potential, we acquired and analyzed spatial transcriptomic data of primary CRC samples and matched liver metastasis samples from public resources. Based on Louvain clustering and spot feature analysis, we classified the spots of the primary tumor samples into clusters 0–9 and the liver metastasis samples into clusters 0–8. Next, we used the SPOTlight tool to project the scRNA-seq data of RIPK2^+^ cluster and cluster 3 onto these spots, with spots exhibiting similar transcriptional profiles being highlighted on the spatial map (Fig. 3A, F). We discovered that cluster 2 of primary tumor tissues corresponded to cluster 3, while the RIPK2^+^ cluster was mapped to cluster 7 (Fig. 3B, C). Cluster 2 and cluster 7 were extracted for further trajectory analysis, which revealed a transition from the less malignant cluster 3 to the more malignant RIPK2^+^ cluster. Spatially, this transition corresponds to deeper tumor invasion (Fig. 3D). Pseudotime analysis further confirmed this finding (Fig. 3E). Then, we similarly projected the RIPK2^+^ cluster onto the liver metastases and found that its corresponding cell cluster were more widespread, including cluster 1, cluster 3, cluster 4, and cluster 8, all of which are tumor cells (Fig. 3G). Additionally, analysis of RIPK2 distribution in this liver metastasis sample similarly indicated that RIPK2 was predominantly expressed in tumor cells (Fig. 3H). Consistent with this, IHC analysis demonstrated that RIPK2 was abundantly overexpressed in tumor cells across three liver metastasis samples (Fig. 3I). Thus, RIPK2^+^ tumor cell cluster possesses highly invasive and metastatic capabilities.

### RIPK2 is a key determinant of CRC metastasis

**Fig. 4:**
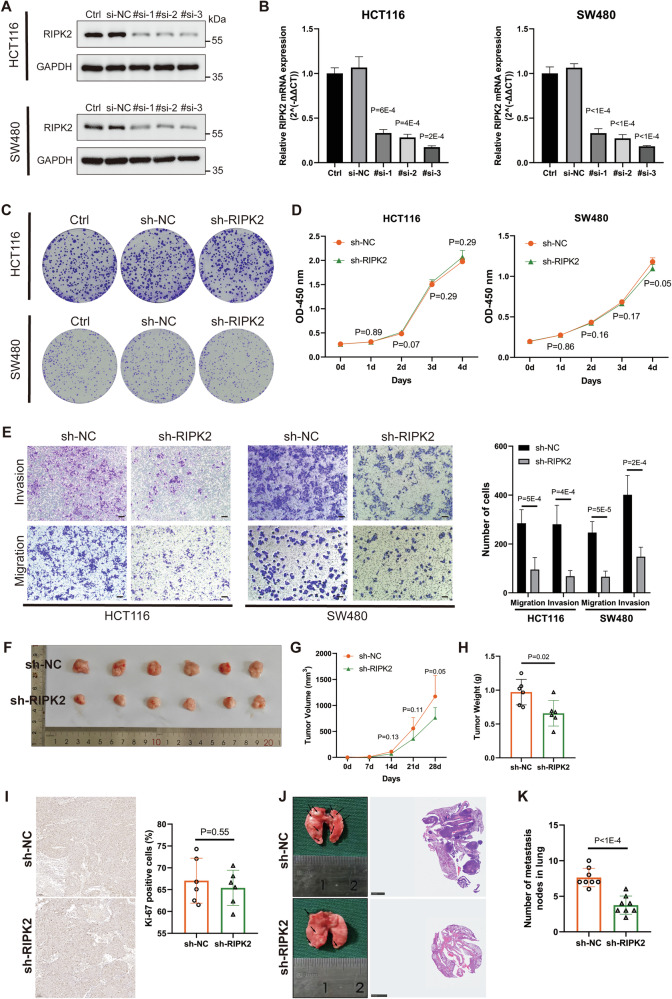
RIPK2 is mainly required for CRC metastasis rather than tumor growth. Efficiency of RIPK2 knockdown with siRNA was detected using western blotting (**A**) and RT-qPCR (**B**), respectively, in HCT116 and SW480 cells. **C** Colony formation assay of cells infected with lentivirus of sh-NC or sh-RIPK2. In addition, we set up a group of lentivirus-free cells. **D** CCK-8 assay of sh-NC or sh-RIPK2 CRC cells. **E** Transwell assay of sh-NC or sh-RIPK2 CRC cells. **F** Representative tumor images in mice bearing HCT116 cells infected with lentivirus of sh-NC or sh-RIPK2. **G** Tumor volume in each group of mice with time. **H** Tumor weight in each group of mice. **I** IHC analysis of Ki-67 level in each group of mice. **J**, **K** For each group of mice, H&E staining to measure the number of tumor nodules in the lung.

To explore whether targeting RIPK2 inhibits CRC progression, we designed three siRNAs to suppress RIPK2 expression in two CRC cell lines, HCT116 and SW480. The third siRNA (#si-3) was selected to construct the corresponding plasmid and lentivirus because it gave the highest gene knockdown efficiency among other siRNAs tested (Fig. 4A, B and Supplementary Fig. 5). RIPK2 knockdown did not significantly affect CRC cells proliferation using colony formation and CCK-8 assays (Fig. 4C, D). Nevertheless, RIPK2 knockdown significantly inhibited CRC cells invasion and migration by transwell assay (Fig. 4E). To verify the above results from in vitro experiments, in vivo experiments were then conducted using xenograft nude mice where subcutaneous and lung metastasis models were respectively established with HCT116 cells infected with either sh-NC or sh-RIPK2 lentivirus. In the subcutaneous tumor model, RIPK2 knockdown resulted in a non-significant reduction in tumor volume (Fig. 4F, G) but significant reduction in tumor weight (Fig. 4H) when compared to the control group. Ki-67 immunohistochemical staining showed no significant difference in Ki-67 levels between the two groups (Fig. 4I). In the lung metastasis model, mice injected with sh-RIPK2-infected cells had significantly fewer metastatic nodules in the lungs compared to the control group (Fig. 4J, K). Taken together, it is concluded that RIPK2 plays a less role in tumor cell proliferation, but a crucial role in regulating the migratory and invasive capabilities of CRC cells.

### DIA proteomics reveals the potential mechanisms of RIPK2 in CRC cells

**Fig. 5:**
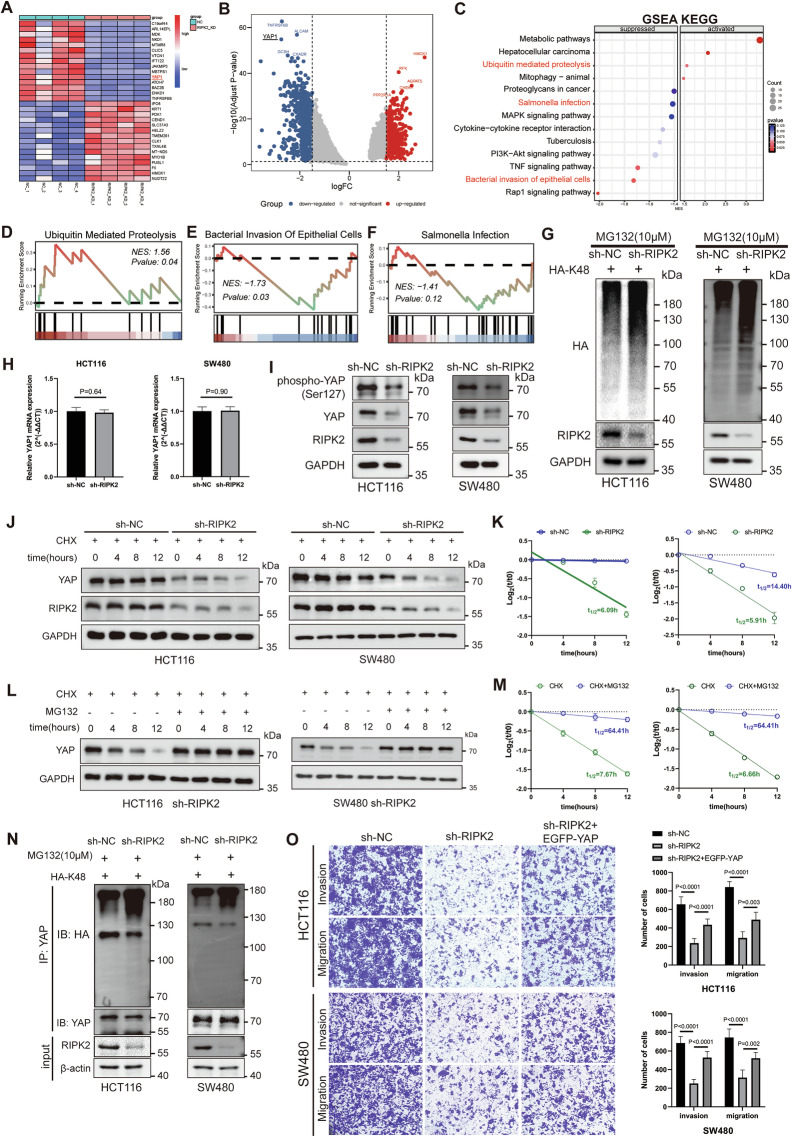
DIA proteomics analysis of the downstream mechanism of RIPK2 in CRC cells. **A** Heatmap of top 30 DEPs in the HCT116 cells with RIPK2 knockdown, compared with negative control cells. **B** Volcano plot of all DEPs using the cut-off criteria of |log2FC | > 1.5 and adjusted *P* value < 0.05. **C** KEGG analysis of significant DEPs by GSEA. GSEA plots showing activation of ubiquitin mediated proteolysis (**D**), inhibition of bacteria invasion of epithelial cells (**E**) and salmonella infection (**F**) in RIPK2-KD HCT116 cells, compared with negative control cells. **G** Ubiquitin (linkage-specific K48) levels in total lysates of sh-NC and sh-RIPK2 CRC cells transfected with HA-K48 plasmids and treated with 10 μM MG132, were analyzed by western blotting. **H** Relative YAP mRNA expression was analyzed by RT-qPCR in CRC cells infected with sh-NC or sh-RIPK2 lentivirus. **I** Representative immunoblots of the indicated proteins of total lysates from HCT116 and SW480 cells infected with sh-NC or sh-RIPK2 lentivirus. Representative immunoblots (**J**) and quantification (**K**) of YAP protein levels in total lysates of sh-RIPK2 HCT116 and SW480 cells, compared with sh-NC CRC cells, in response to CHX treatment. Representative immunoblots (**L**) and quantification (**M**) of YAP protein levels in total lysates of sh-RIPK2 HCT116 and SW480 cells treated with DMSO or MG132, in the presence of CHX treatment. **N** YAP protein was immunoprecipitated from sh-NC or sh-RIPK2 HCT116 and SW480 cells transiently transfected with 10 μg HA-K48 plasmids and treated with 10 μM MG132. Immunoprecipitation product and total lysates were analyzed by western blotting. **O** Transwell assay was used to evaluate whether reintroducing YAP could restore the migratory and invasive capabilities of HCT116 and SW480 cells.

To investigate how RIPK2 influences CRC cell metastasis, we performed proteomic analysis to examine regulation of protein expressions by RIPK2. 5554 protein groups with FDR < 1% were identified. Using cut-offs of |log2FC | >1.5 and *p* value < 0.05, 1557 proteins were found to be significantly downregulated, while 159 were significantly upregulated in the RIPK2 knockdown cells compared to the control groups (Fig. 5A, B).

Next, these differentially expressed proteins (DEPs) were further subjected to GSEA analysis. GO showed significant inhibition of cell migration and cell motility in biological process, suppression of transcription regulator complex in cellular component, and repression of transcription factor binding in molecular function (Supplementary Fig. 6). KEGG showed significant activation of ubiquitin mediated proteolysis and inhibition of bacteria invasion of epithelial cells and salmonella infection (Fig. 5C–F). This result is similar to the GSEA analysis in the RIPK2^+^ cluster from scRNA-seq data (Fig. 2L–N). In conclusion, RIPK2 knockdown not only suppressed transcriptional regulation and bacteria-driven signaling pathways, but also activated ubiquitin-mediated proteasomal degradation.

### RIPK2 prevents YAP from proteasomal degradation

Remarkably, classical oncoprotein YAP was among the 1557 significantly downregulated proteins (Fig. 5A, B). YAP has long been linked in the progression of various human cancers and recognized to be the central transcription control gene of the Hippo pathway [29]. However, YAP mRNA level was not affected by RIPK2 knockdown (Fig. 5H). Additionally, both YAP phosphorylation levels and protein abundance were significantly suppressed by RIPK2 inhibition (Fig. 5I). Combined with GSEA analysis of proteomics, we suspect that YAP protein was degraded via the ubiquitin-mediated proteasomal pathway. We uncovered the increase of total K48-linked ubiquitination levels in CRC cells following RIPK2 knockdown (Fig. 5G). The proteasomal degradation of YAP in the RIPK2 knockdown cells was further probed by adding a protein synthesis inhibitor, cycloheximide (CHX), in the RIPK2 knockdown cells at a time course of 0 h, 4 h, 8 h, and 12 h. It was found the half-life of YAP protein was significantly reduced compared to the same RIPK2 knockdown cells without the inhibitor (Fig. 5J, K). When proteasome inhibitor MG132 was added into the cells, the YAP protein level was recovered as that without any inhibitor in the cells (Fig. 5L, M). To assess whether YAP is degraded via the K48 ubiquitin pathway, ubiquitination assays were performed. The result showed that YAP K48-linked ubiquitination levels increased significantly after RIPK2 knockdown (Fig. 5N), indicating that YAP is degraded indeed via the K48 ubiquitin pathway. Consistently, immunofluorescence and nuclear-cytoplasmic fractionation assays also confirmed the findings (Supplementary Fig. 7). However, neither the protein levels nor the nuclear localization of YAP was affected by RIPK2 overexpression (Supplementary Fig. 7). It is to note that although the YAP stability was controlled by RIPK2, the nuclear localization of YAP was not affected with or without RIPK2. To explore further, we reintroduced YAP expression in RIPK2 knockdown cells and observed whether the migratory and invasive abilities of CRC cells were restored. The results showed that CRC cell migration and invasion were not fully restored (Fig. 5O), suggesting that YAP may not be the only one downstream effector of RIPK2.

### E3 ubiquitin ligase ITCH is a key regulator of RIPK2’s effect on YAP stability, being associated with MDP

**Fig. 6:**
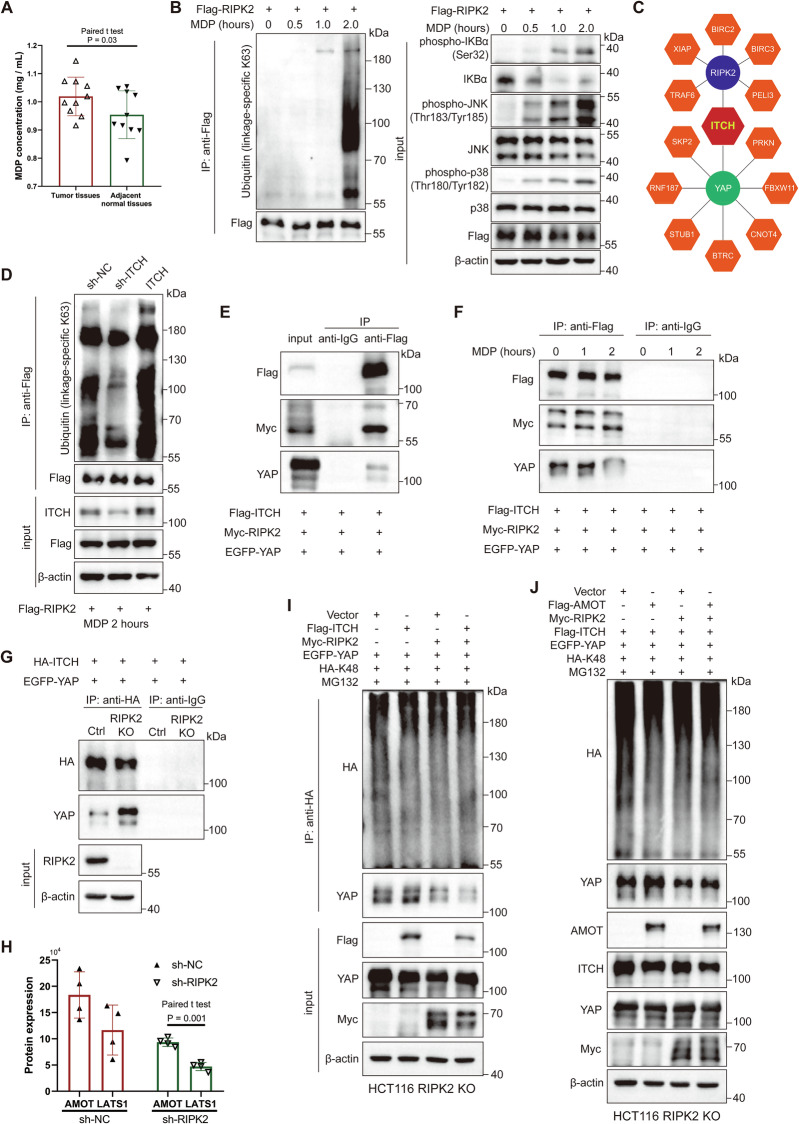
E3 ubiquitin ligase ITCH is a key regulator of RIPK2’s effect on YAP stability, being associated with MDP. **A** Bar plot of MDP concentration in CRC tumor tissues, compared with paired adjacent normal tissues. **B** HCT116 cells transfected Flag-RIPK2 plasmids were stimulated with MDP for 0, 0.5, 1, and 2 h. Their lysates were immunoprecipitated with indicated Flag antibody, and precipitates and lysates were examined by western blotting using indicated antibodies. **C** Schematic representation of the reported E3 ubiquitin ligases involved in the regulation of RIPK2 or YAP. **D** HCT116 cells transfected with Flag-RIPK2 plasmid were stimulated with MDP for 2 h following ITCH expression modulation (including knockdown and overexpression). Their lysates were immunoprecipitated with indicated Flag antibody, and precipitates and lysates were examined by western blotting using indicated antibodies. **E** HCT116 cells were co-transfected with Flag-ITCH, Myc-RIPK2, and EGFP-YAP plasmids. The interactions between ITCH and RIPK2 or YAP were confirmed using a Co-IP assay. **F** HCT116 cells, co-transfected with Flag-ITCH, Myc-RIPK2, and EGFP-YAP plasmids, were stimulated with MDP for 0, 1, and 2 h. The changes in the interactions between ITCH and RIPK2 or YAP were analyzed using a Co-IP assay. **G** The interaction between ITCH and YAP was analyzed by Co-IP before and after RIPK2 knockout in HCT116 cells. **H** Bar plot of AMOT and LATS1 protein expression in proteomics of HCT116 cells treated with NC and RIPK2 knockdown. **I** The ubiquitination of YAP was assessed using a K48 ubiquitin pull-down assay to determine whether it is influenced by ITCH expression in RIPK2-KO HCT116 cells. Following this, RIPK2 was reintroduced, and the impact of ITCH expression on YAP ubiquitination was re-evaluated. **J** Using K48 ubiquitin pull-down, the ubiquitination of YAP was assessed to determine if it is affected by AMOT expression in RIPK2-KO HCT116 cells. Following RIPK2 reintroduction, YAP ubiquitination was reassessed to see if AMOT expression still influences this process.

To explore the mechanism by which RIPK2 stabilizes YAP, we first confirmed whether RIPK2 directly interacts with YAP to stabilize its expression. CO-IP experiments showed that there was no significant interaction between the two in either SW480 or HCT116 cells (Supplementary Fig. 8). However, both GSEA analyses of RIPK2^+^ cluster and proteomics following RIPK2 knockdown pointed to significant activation of bacteria invasion of epithelia cells, suggesting a strong relation between RIPK2 activation and bacteria invasion (Figs. 2N, 5E). Notably, the intracellular receptor NOD2 responds to MDP, a breakdown product of bacterial peptidoglycan, mediating K63-linked polyubiquitination of RIPK2, thereby activating NF-κB and MAPK signaling pathways [30–32]. Thus, we hypothesized that RIPK2 underwent K63-linked ubiquitination in response to MDP stimulation in CRC cells, and this process could be closely related to YAP stability. Firstly, we measured MDP concentration in 10 pairs of CRC tumor tissues and matched adjacent non-cancerous tissues, uncovering that MDP levels were significantly higher in tumor tissues compared to normal tissues (Fig. 6A). Next, we stimulated HCT116 cells with MDP for 0, 0.5, 1, and 2 h, observing that after 2 h of MDP stimulation, RIPK2 exhibited pronounced K63-linked polyubiquitination, and there was significant activation of IκBα, JNK, and p38 within the cells (Fig. 6B). The activation of these signaling pathways is well-known to be associated with tumor metastasis [33, 34].

To determine whether RIPK2 polyubiquitination affects the proteasomal stability of YAP, we first investigated whether they share a common ubiquitin ligase regulator, due to the fact that there is no direct interaction between RIPK2 and YAP. Among the reported ubiquitin ligases, we found that only ITCH is capable of directly regulating both K63-linked ubiquitination of RIPK2 and ubiquitination of YAP [20–22] (Fig. 6C). ITCH knockdown significantly inhibited K63-linked ubiquitination of RIPK2, while ITCH overexpression significantly enhanced it in HCT116 cell line (Fig. 6D). Then, we used CO-IP assay to confirm the interaction between ITCH and both RIPK2 and YAP. Moreover, ITCH binds more strongly to RIPK2 than to YAP when equal amounts of protein were loaded in HCT116 cell (Fig. 6E). Furthermore, when HCT116 cells were stimulated with MDP, the binding between ITCH and RIPK2 showed a gradual increase, while the binding with YAP significantly decreased after 2 h of stimulation (Fig. 6F). These results indicated that MDP stimulation not only promotes RIPK2 ubiquitination to activate NF-κB and MAPK signaling pathways, but also enhances the binding of ITCH to RIPK2, thereby protecting YAP stability.

To investigate whether ITCH targets YAP in the absence of RIPK2, we stably knocked out RIPK2 from HCT116 cell line using the CRISPR/Cas9 system, allowing us to better study the relationship between ITCH and YAP in CRC. Compared with the control group, the binding between ITCH and YAP significantly increased in the RIPK2 knockout (RIPK2-KO) group (Fig. 6G), indicating that in the absence of RIPK2, ITCH could directly target YAP, promoting its degradation in HCT116 cells. However, YAP ubiquitination by ITCH largely depends on the competitive interaction between AMOT130 and LATS1/2 for ITCH. Specifically, in the presence of stable AMOT, ITCH promotes YAP degradation, while in the absence of AMOT, ITCH instead promotes the degradation of LATS1, leading to YAP activation [21, 22, 35]. Consequently, our proteomic analysis revealed that, regardless of RIPK2 knockdown, the abundance of AMOT protein in HCT116 cells was significantly higher than that of LATS1 (Fig. 6H). To further determine whether ITCH-mediated ubiquitination of YAP in CRC cells is related to RIPK2 and AMOT, we established an in vitro RIPK2-KO cell model of YAP K48-linked ubiquitination. Consistently, by performing a pull-down assay with exogenous K48 ubiquitin, we found that ITCH overexpression significantly increased the ubiquitination level of YAP, regardless of AMOT overexpression (Fig. 6I, J). However, ITCH no longer ubiquitinated YAP, even in the presence of AMOT, when RIPK2 was reintroduced (Fig. 6I, J). This indicated that ITCH preferentially binds to RIPK2, promoting its K63-linked ubiquitination while simultaneously protecting YAP protein levels and maintaining its nuclear localization in CRC cells. Together, these processes contribute to the promotion of CRC cell metastasis.

### Pharmacological inhibition of RIPK2 promotes YAP degradation by K48-linked ubiquitination

**Fig. 7:**
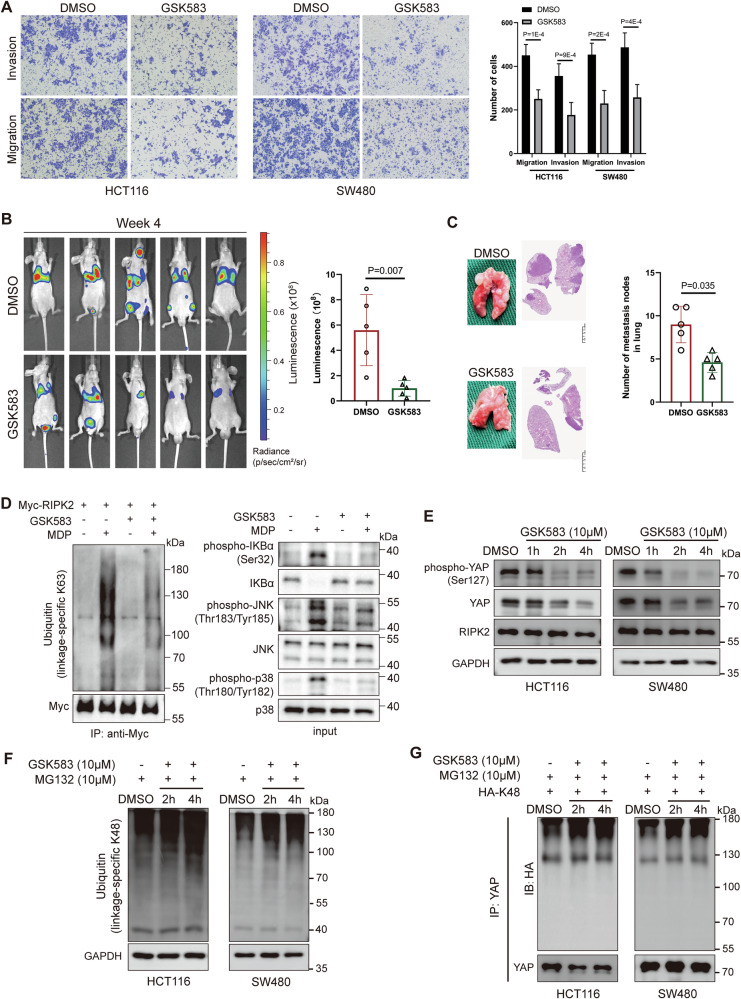
GSK583 inhibits CRC metastasis and promotes YAP degradation by K48-linked ubiquitination. **A** Transwell assay of CRC cells treated with DMSO or GSK583. **B** Luminescence images (Right) and bar plot (Left) of mice treated with GSK583, compared with control treatment. **C** H&E staining (Left) and Bar plot (Right) of measuring effect of GSK583 treatment on the number of pulmonary metastases in mice. **D** HCT116 cells were treated with MDP for 2 h in the presence or absence of GSK583 (added 2 h prior). Their lysates were immunoprecipitated with indicated RIPK2 antibody, and precipitates and lysates were examined by western blotting using indicated antibodies. **E** Representative immunoblots of the indicated proteins of total lysates from HCT116 (left) and SW480 (right) cells treated with 10 μM GSK583 at 1 h, 2 h, and 4 h, compared with control cells treated with DMSO. **F** Ubiquitin (linkage-specific K48) levels in total lysates of CRC cells transfected with HA-K48 plasmids and treated with GSK583 and MG132, compared with control cells treated with DMSO and MG132. **G** YAP protein was immunoprecipitated from CRC cells transiently transfected with HA-K48 plasmids and treated with GSK583 and MG132. Immunoprecipitation product were analyzed by western blotting.

We have shown that RIPK2 played a crucial role in CRC via positively regulating YAP through Itch. This identification helps find an effective inhibitor of RIPK2 in targeted therapy for CRC treatment. A highly potent, orally active, and selective inhibitor of RIPK2, GSK583, was first selected for the effectiveness of RIPK2 inhibition. To determine whether GSK583 diminishes the metastatic potential of CRC cells, we conducted in vitro transwell assays and in vivo lung metastasis models in nude mice. GSK583 significantly suppressed the migration and invasion abilities of HCT116 and SW480 cells (Fig. 7A). In the in vivo experiment, we injected luciferase-expressing HCT116 cells into the tail veins of nude mice and waited for a week to allow for the formation of early lung metastatic lesions. Then, these mice were randomly divided into two groups: a control treatment group and a group treated with GSK583 daily. Consistent with the results of RIPK2 knockdown, GSK583 significantly reduced the luminescence intensity of lung metastases (Fig. 7B), which largely correspond to lung metastasis as supported by histopathological analysis (Fig. 7C).

Moreover, in a similar fashion to ITCH knockdown (Fig. 6D), treatment of HCT116 cells with GSK583 led to a strong reduction in MDP-stimulated RIPK2 ubiquitination, as well as NF-κB and MAPK activation (Fig. 7D). Then, the effectiveness of GSK583 was further examined using the cells treated with 10 μM GSK583 at 0 h, 1 h, 2 h, and 4 h, respectively. The result showed that a significant reduction in the expression of YAP protein and its phosphorylation occurred around 2 h of the treatment (Fig. 7E). In a parallel experiment, the cells were co-treated with GSK583 and proteasome inhibitor MG132 at the same time course as above, i.e., 0 h, 2 h, and 4 h, respectively. This co-treatment resulted in a time-dependent increase in overall K48-linked ubiquitination levels (Fig. 7F). A further introduction of a K48 plasmid in the cells showed that the K48-linked ubiquitination of YAP protein significantly increased after 2 h of GSK583 treatment (Fig. 7G). The above results indicate that GSK583 not only inhibits CRC migration and invasion, but also promotes protein degradation pathways. Thus, this inhibitor can be a very promising inhibitor of RIPK2 in CRC treatment.

## Discussion

RIPK2 is a Ser/Thr/Tyr protein kinase, which was first identified in 1998 [36]. It contains two conserved domains: an N-terminal kinase domain and a C-terminal caspase activation and recruitment domain (CARD) that mediates the recruitment of CARD-containing proteins, and functions mainly in inflammation and innate immunity via the NOD/RIPK2/NF-κB signaling pathway [16]. Recent studies showed that RIPK2 was also involved in a number of cancers, such as pancreatic cancer [37], breast cancer [38], hepatocellular carcinoma [39], prostate cancer [40], and so on. However, RIPK2’s role in CRC remains controversial. Couturier-Maillard A et al. suggested that loss of RIPK2 led to a proinflammatory microenvironment, which enhances epithelial dysplasia following chemically induced damage, and this may be one of the key mechanisms through which inflammatory bowel disease progresses to CRC [24]. Conversely, Garo LP et al. found that activation of RIPK2 by MDP stimulation in myeloid cells leads to the release of IL-17 promoting cytokines, which in turn activates tumorigenic IL-17R signaling in intestinal epithelial cells [25]. Based on our single-cell transcriptomic analysis, we think this is likely related to specific cell types. Additionally, it’s important to note that both of these studies are based on research linking colorectal cancer to inflammatory bowel disease (IBD). Thus, to better understand RIPK2’s role in CRC, we expanded our research beyond the context of IBD.

In this study, by adopting various strategies and techniques, we successfully identify RIPK2, its strong association with poor prognosis of CRC and its critical role in CRC metastasis. Our findings align with previous findings of Sang et al. in pancreatic cancer [37] and Yan et al. in prostate cancer [40]. Moreover, MDP derived from bacterial breakdown products can directly stimulate K63-linked ubiquitination of RIPK2, followed by the activation of NF-κB and MAPK signaling pathways. Within this classical mechanism, we surprisingly discovered a competitive interaction between the ubiquitination mechanism of RIPK2 and that of the Hippo signaling pathway, with the E3 ubiquitin ligase ITCH serving as a key regulator. This discovery has never been reported before and adds valuable insight into the molecular mechanism by which RIPK2 promotes tumor progression.

ITCH is a member of E3 ubiquitin ligase family which contains over 700 members that conjugate ubiquitin to target proteins, resulting in an array of cellular responses, including DNA repair, pro-survival signaling and protein degradation [41]. Structurally, it contains a C2 domain, proline-rich region, WW domains, HECT domain and amino acid region for phosphorylation [42]. It is intriguing that ITCH, beyond its role in catalyzing K48-linked polyubiquitination for proteolysis, also contributes to ubiquitin chain diversity by assembling K63, K27, and K33-linked polyubiquitin chains [43]. This is similar to our findings that ITCH not only promotes K63-linked ubiquitination of RIPK2 but also regulates the K48-linked ubiquitination of YAP. The mechanism by which ITCH is involved in YAP ubiquitination is very complicated, being regulated not only by deubiquitinating enzymes but also by the competitive interaction between AMOT and LATS1 [21, 22, 44, 45], which determines how ITCH modulates YAP. This could potentially lead to heterogeneity among tumor cells. In any case, our study demonstrated that the ubiquitination function of RIPK2, mediated by ITCH, was indispensable for stabilization of YAP in mCRC.

It was noted that reintroducing YAP did not fully rescue the migratory and invasive abilities of CRC cells with RIPK2 knockdown. This precisely indicates that RIPK2 exerts a powerful influence in CRC cells, with effects that extend beyond a single downstream effector like YAP. Additionally, this study emphasizes the ubiquitination function of RIPK2, which is a primary role of its CARD domain [46, 47]. However, the kinase function of RIPK2 has yet to be unclarified, primarily because its direct substrates remain poorly defined. Whether the activation of its kinase function is related to bacterial invasion (especially MDP), or whether it can promote tumor metastasis, remains unknown and warrants further investigation. Regardless, RIPK2 is a particularly attractive target for cancer therapy.

In conclusion, we have identified a crucial role of RIPK2 in CRC for the first time, and also identified its function mechanism via stabilization of YAP in cancers for the first time. In addition, an effective pharmacological inhibitor, GSK583, to prevent CRC development is also demonstrated. Our study provides the potential opportunity for future CRC treatment.

## Supplementary information

Supplementary materials & methods

Supplementary Figures

Additional Supplementary Files

Original western blots

## Data Availability

All datasets related to the bioinformatics analyses have been described within Supplementary materials and methods. The proteomics data generated in this study have been deposited to the ProteomeXchange Consortium (https://proteomecentral.proteomexchange.org) via the iProX partner repository [48, 49] with the dataset identifier PXD061372.
