# Supplementary material for: RIPK2 promotes colorectal cancer metastasis by protecting YAP degradation from ITCH-mediated ubiquitination
Source: Cell Death Dis. 2025 Apr 4;16(1):248. doi: 10.1038/s41419-025-07599-9 (PMC11971272; doi:10.1038/s41419-025-07599-9)
Supplement: Supplementary file 1 — Supplementary materials & methods [file 41419_2025_7599_MOESM1_ESM.docx]

**Supplementary materials & methods**

**Bulk RNA-seq analysis**

To better compare the differential genes between CRC tumor and adjacent tissues, we screened some datasets with one-to-one matching samples from public databases, such as Gene Expression Omnibus (GEO) database, TCGA database and Clinical Proteomic Tumor Analysis Consortium (CPTAC) database. In GEO database and TCGA database, four CRC cohorts (GSE22598, n=17; GSE39582, n=17; COAD, n=41; READ, n=9) were analyzed to screen for differential genes by selecting cut-off values of |log2fold change (FC)| > 1.0 and adjusted p-value < 0.05. Then, Kinbase database (http://www.kinase.com/), holding information on protein kinase genes found in human genomes^1^, was used to filter out non-protein kinase genes. GSE41258 (n=44) was used to validate the differential expression of kinase genes at mRNA level, and CPTAC database (PDC000116, n=96) was used to examine their differential expression at protein level. Due to lack of clinical information in GSE22598, to further expand the sample size, one cohort (GSE87211, n=160) was added to analyze for OS using Cox regression. The information summarized from five cohorts (GSE39582, GSE41258, GSE87211, COAD and READ) can be found in **Additional Supplementary Files 2**.

**ScRNA-seq analysis**

***Data collection and preprocessing***. We also screened the datasets containing paired tumor and adjacent normal samples, GSE231559 and GSE245552 (detailed in **Additional Supplementary Files 3**), from GEO database. Quality control was performed using four criteria: (1) filtering cells with detected genes between 300 and 5000, and excluding the top 3% of cells with the highest unique molecular identifiers (UMIs); (2) ensuring mitochondrial gene UMIs were less than 30%; (3) setting the hemoglobin gene percentage between 0-3%; (4) ensuring ribosomal gene UMIs were less than 50%. Samples from patient Pt17, which had a low proportion of tumor cells, were excluded due to poor data quality. To eliminate doublets, DoubletFinder (v2.0.4) was employed with a doublet formation rate of 7.5%^2^. Post-filtering, remaining cells were analyzed using Seurat (v4.4.0) for dimensionality reduction and clustering^3^. Gene expression was normalized and scaled using NormalizeData and ScaleData functions, respectively. The top 3000 variable genes were selected using the FindVariableFeatures function for principal component analysis (PCA). Clustering was performed using the FindClusters function with the first 20 principal components, resulting in 18 clusters. Harmony (v1.2.0) was used to remove batch effects and integrate cells. Visualization was conducted using the t-distributed stochastic neighbor embedding (tSNE) algorithm^4^. Differential gene expression analysis was performed using the FindAllMarkers function in Seurat with the Wilcoxon test. Genes expressed in more than 10% of cells in each cluster with a log fold change greater than 0.25 were identified as differentially expressed. The top 10 differentially expressed genes per cluster were annotated using the CellMarker database and visualized using the FeaturePlot function in Seurat. Detailed information on marker genes for various cell types is provided in **Additional Supplementary Files 4**.

***InferCNV analysis*.** Chromosomal copy number variations (CNVs) were estimated using inferCNV (v1.3.3), with endothelial cells as the reference^5^. Initial CNV scores for each cell were calculated and adjusted relative to endothelial cells. Amplifications and deletions were visualized in red and blue, respectively, with intensity indicating the degree of CNV.

***Pseudotime analysis*.** Monocle2 (v2.3.0) was used to construct single-cell pseudotime trajectories, revealing cellular state transitions^6^. Genes defining the trajectory were selected using the dispersionTable function, and dimensionality was reduced using the DDRTree method. The plot_cell_trajectory function visualized the minimum spanning tree of cells. CytoTRACE (v0.3.3) was used to predict differentiation scores, which were then mapped onto the monocle pseudotime trajectory^7^.

***Gene Set Variation Analysis (GSVA)*.** GSVA (v1.5.0) was used to compare enrichment of 50 hallmark pathways between cancerous and adjacent non-cancerous epithelial cells, with differential pathway analysis performed using the limma package. Pathways with an absolute t-value greater than 2 were considered significantly different and visualized using the ggplot2 package.

***Gene set enrichment analysis (GSEA)***. The clusterProfiler package was used to conduct GSEA, including Kyoto encyclopedia of genes and genomes (KEGG) and gene ontology (GO) that contains biological process (BP), cellular component (CC) and molecular function (MF). Pathways were visualized using GseaVis package.

**Spatial transcriptomics (ST) analysis**

We acquired ST data of primary CRC and liver metastasis lesions from the study by Wu et al^8^. The Seurat package was utilized to process ST data, generating a gene-spot matrix. Spots with fewer than 10 detected genes were filtered out to ensure data quality. We employed SCTransform for data normalization, followed by dimensionality reduction using the RunPCA function. Clustering of ST spots was performed using the FindNeighbors and FindClusters functions, and the data were visualized with RunUMAP. Given that each spot contains multiple cell types, we combined the scRNA-seq data and predicted main cell types by using SPOTlight package (v1.5.1)^9^.

The SpatialFeaturePlot function was used to assess the potential expression levels between different subclusters. High-expression regions were then extracted for further trajectory analysis. We employed the stLearn package (v0.4.12) to infer pseudo-spatiotemporal distances (PST)^10^. Dimensionality reduction was performed using the st.em.run_pca function, and spatial data were normalized with SME_normalize. To enhance the resolution, Louvain clustering was applied to integrate cell type predictions from SPOTlight into the stLearn framework, ultimately constructing spatial trajectories. Spatial trajectories were further constructed and visualized using st.pl.cluster_plot function. Additionally, we used Monocle2 to construct pseudotime trajectories of ST.

**SiRNAs, plasmids and lentiviruses**

Three siRNAs’ sequences targeting RIPK2 (si-RIPK2) were designed and produced from Proteinbio Corporation (Nanjing, China). The sequence of si-ITCH was acquired from the study by Lim SK et al^11^. The siRNA sequence with the highest efficiency was cloned into the PGMLV-mScarlet-puro vector to construct lentivirus. The full length of RIPK2 was cloned into the PGMLV-CMV-puro vector with Flag-tag or Myc-tag. The lentiviruses and the above plasmids were generated by Genomeditech (Shanghai, China). The plasmids expressed ITCH with HA-tag (#P53060) or Flag-tag (#P33655) and expressed AMOT with Flag-tag (#P38083) were purchased from MIAOLING biology Corporation (Wuhan, China). The sequences of siRNAs are listed as follows (5’-3’):

RIPK2, #si-1, CACCAAUCCUUUGCAGAUAAUTT;

RIPK2, #si-2, GCACGUAUGAUCUCUCUAAUATT;

RIPK2, #si-3, CCAUGCUCUUCAGCAAUAAUATT;

ITCH, siRNA, CAAUUUGUCUUGAUGGGCUACAGUU;

Control, si-NC, UUCUCCGAACGUGUCACGUTT.

**RIPK2 knockout by CRISPR/Cas9**

Three sequences of sgRNAs targeting RIPK2 were designed and produced by MIAOLING biology Corporation. Prepare ribonucleoprotein (RNP) complexes by combining sgRNAs and Cas9 at a 5:1 to 10:1 ratio. Perform nuclear transfection of RNPs using an electroporator (Thermo Scientific) according to the manufacturer’s instructions. Following electroporation, conduct single-cell sorting and expansion, and verify the genotype using Sanger sequencing. The sequences of sgRNAs are listed as follows (5’-3’):

RIPK2, #sgRNA-1, GCCCGGGACCATGAACGGGG

RIPK2, #sgRNA-2, TGGACGCGATGGACTCGGCG

RIPK2, #sgRNA-3, ACTTCGTGGACGTGTAGGTG

**Animal studies**

***Subcutaneous xenograft model***. To optimize both statistical rigor and resource allocation, a sample size of 6 subjects per group was selected for the experimental design. Firstly, HCT116 cells with negative control (NC) or RIPK2 knockdown (RIPK2-KD) lentivirus were adjusted to 1×10^7^/ml. Then, the 12 mice were randomly divided into the NC group and RIPK2-KD group. 200 μL cell suspension was subcutaneously injected into the left flank of each mouse. Next, tumor size of each mouse was measured every 7 days with a caliper. Finally, all mice were euthanized, and their subcutaneous tumor tissues were collected for weighting and volume measurement. The tumor volume was calculated using the formula of (width^2^×length)/2.

***Metastatic model****.* Based on a balance between statistical significance and experimental costs, total of 16 mice were randomly assigned to two groups (n = 8 per group). For each mouse, 100 μL of 1×10^6^ cells with NC or RIPK2 KD lentivirus in PBS was slowly injected into their tail vein to construct the metastatic model (mainly lung metastasis). After around thirty-five days, all mice were euthanized, and their lungs were dissected and isolated, and then fixed in 4% paraformaldehyde for further analysis of hematoxylin and eosin (H&E) staining.

For drug treatment, after cell suspension with luciferase expression was injected a week later, we divided all mice into two groups (DMSO vs. GSK583) randomly (n = 5 per group). Mice were administrated GSK583 (10mg/kg) (#HY-100339, MedChemExpress (MCE), Shanghai, China) or DMSO orally via gavage once a day for a month. After 4 weeks, tumor metastasis burden of each mouse was evaluated using in vivo imaging technology.

**DIA proteomics**

Each sample of cells infected with NC or RIPK2-KD lentivirus was harvested and lysed with 200 μL 8M urea containing protease inhibitor (#S8830, Sigma Aldrich), and protein concentration was determined. Proteins were reduced with DT-Dithiothreitol (DTT), alkylated with iodoacetamide (IAM), and digested overnight with trypsin. Peptides were desalted using SoLAμ HRP 2mg/mL 96-well plate (#60209-001, Thermo Scientific) and dried. LC-MS/MS analysis was conducted on an Ultimate^TM^ 3000RSLC system (Thermo Scientific) coupled with a Q Exactive HF-X mass spectrometer (Thermo Scientific) in DIA mode. Peptides were separated using a 160-minute gradient that mobile phase B (0.1% formic acid in 80% acetonitrile) was from 1% to 8% in 4 minutes, from 8% to 30% in 141 minutes, from 30% to 90% in 5 minutes, from 90% to 1% in 1 minutes, 1% in 9 minutes. Full MS scans were acquired from 350-1200 m/z, followed by 80 DIA windows. Raw data were imported into DIA-NN (v1.8.0) targeted extracted with predict human proteomics database, and the remaining parameters were controlled by default to control FDR < 1% of peptide and protein levels with default parameters^12, 13^. Output the protein intensities calculated by DIAN from the mean of the top3 peptides. This protein intensity was imported into Perseus and Metaboanalyst (https://www.metaboanalyst.ca/) for statistical analysis. Then, remove proteins with missing values greater than 50%, and fill missing values in the remaining proteins using the K-Nearest Neighbor (KNN) method. For statistical comparison, all protein values were log_2_-transformed, and identified as differentially expressed proteins with |log2fold change (FC)| > 1.5 and adjusted p-value < 0.05. Finally, these differentially expressed proteins were analyzed for GSEA. Details of all differentially expressed proteins are provided in **Additional Supplementary Files 5**.

**Co-IP assay**

Cell protein was collected using IP lysis buffer (#87787, Thermo Scientific). Then, 500 μL of 2 mg/mL protein supernatant was incubated with the primary antibody or IgG overnight at 4 ℃, and the antigen sample/antibody mixture was incubated with 0.2 mg pre-washed protein A/G magnetic beads (#88802, Thermo Scientific) at room temperature for 2 hours. After washing beads for three times using magnetic stand (#12321D, Thermo Scientific), bound proteins were eluted and analyzed by western blotting as indicated.

**K48-ubiquitination assay**

Before cell protein was extracted using IP lysis buffer, cells were transfected with plasmid of pCMV-HA-ubiquitin-K48 (#P31802, MIAOLING Biology) treated with 10 μM MG132 (#HY-13259, MCE) for 6 hours. Then, the following steps are consistent with CO-IP assay.

**MDP Concentration measurement**

The MDP concentration of CRC and paired normal tissues (n = 10, respectively) was examined as previous described^14, 15^. Briefly, 100 mg of each sample was homogenized with 1 mL chloroform/methanol/0.1 N HCl, 1:2:0.8 vol/vol/vol, and centrifuged at 8000 × g for 20 min. The aqueous phase was re-extracted with chloroform and 0.1 N HCl, followed by centrifugation. The final aqueous phase was evaporated under vacuum, dissolved in water, and analyzed via high-performance liquid chromatography (HPLC) using a reverse-phase Li-Chrosphere column. Acetonitrile (95:5) was the solvent, flowing at 1 mL/min. Additionally, samples were spotted on silica gel thin-layer chromatography plates, developed with a methylene chloride/methanol/water/acetic acid solvent system, and detected using iodine vapor. Bands corresponding to standard MDP were eluted, treated with periodic acid, and reacted with 2-thiobarbituric acid in acidic butanol. Absorbance was measured at 549 nm, with a calibration curve created using N-acetylmuramic acid.

**Immunofluorescence (IF)**

After 500μL (1.5×10^4^) cells were seeded into a 24-well plate with cell coverslips overnight, they were transfected with the 20 pmol siRNA (si-NC or si-RIPK2) or 0.5 μg plasmid expressed RIPK2 for 48 hours, followed by fixing cells in 4% paraformaldehyde, permeabilizing cells with 0.3% Triton X-100 and blocking cells with normal goat serum (#SL038, Solarbio, Beijing, China). The coverslips were incubated with primary antibodies at 4 ℃ overnight. Then, the secondary antibodies incubated for 2 hours at room temperature in the dark, followed by staining coverslips with DAPI (#C1006, Beyotime). Finally, the stained cell coverslips were imaged by confocal laser scanning microscope (NIKON Eclipse Ti, Japan).

**Kaplan–Meier survival analysis**

In public databases, we used five cohorts (GSE39582, GSE41258, GSE87211, COAD and READ) to perform Kaplan–Meier survival analysis for OS. According to the result of IHC, survival analyses for OS and recurrence-free survival (RFS) were performed using information of 152 CRC patients we collected. Log-rank test was used to compute hazard ratios (HR) and statistical significance of difference between groups by R software (v4.2.2).

**Gene-specific primers (5’-3’)**

| Genes | Forward sequence (5’ – 3’) | Reverse Sequence (5’ – 3’) |
| --- | --- | --- |
| RIPK2 | CTACCACAAACTCGCCGACC | ACATCCTTTCTTTCACTGTCGAG |
| YAP1 | GCTACAGTGTCCCTCGAACC | TCCTTCCAGTGTTCCAAGGT |
| GAPDH | TCAACGGATTTGGTCGTATTG | TGGGTGGAATCATATTGGAAC |

**Antibodies**

| Antibody | Manufacturer | Catalog number | Application | Dilution |
| --- | --- | --- | --- | --- |
| RIPK2 | CST | #4142 | WB | 1:1000 |
|  | Santacruz | #sc-166765 | IP | 1:30 |
|  | Abcam | #ab8428 | IF | 1:50 |
|  | Origene | #TA332524 | IHC | 1:900 |
| YAP | CST | #14074 | WB | 1:2000 |
|  |  |  | IF | 1:100 |
|  | Santacruz | #sc-376830 | IP | 1:50 |
| phosopho-YAP (Ser127) | CST | #13008 | WB | 1:1000 |
| ubiquitin (K63-linkage specific) | Abcam | #ab179434 | WB | 1:2000 |
| IκBα | CST | #4814 | WB | 1:2000 |
| phosopho-IκBα (Ser32) | CST | #2859 | WB | 1:1000 |
| JNK | CST | #9252 | WB | 1:2000 |
| phosopho-JNK (Thr183/Tyr185) | CST | #9251 | WB | 1:1000 |
| p38 | CST | #9212 | WB | 1:2000 |
| phosopho-p38 (Thr180/Tyr182) | CST | #9211 | WB | 1:1000 |
| ITCH | Proteintech | #20920-1-AP | WB | 1:5000 |
| AMOT | Proteintech | #24550-1-AP | WB | 1:5000 |
| HA | Sigma | #H9658 | WB | 1:5000 |
|  |  |  | IP | 1:200 |
| Flag | Sigma | #F1804 | WB | 1:5000 |
|  |  |  | IP | 1:200 |
| Myc | Proteintech | #60003-2-Ig | WB | 1:5000 |
|  | Sigma | #05-724 | IP | 1:200 |
| GAPDH | Abcam | #ab8245 | WB | 1:5000 |
| β-actin | Sigma | #A5441 | WB | 1:5000 |
| Ki-67 | Servicebio | #GB121499 | IHC | 1:500 |
| Goat anti-rabbit IgG | SAB | #L3012 | WB | 1:8000 |
| Goat anti-mouse IgG | SAB | #L3032 | WB | 1:8000 |
| Alexa Fluor® 594-conjugated Goat Anti-Mouse IgG | Abcam | #ab150116 | IF | 1:200 |
| Alexa Fluor® 488-conjugated Goat Anti-Rabbit IgG | Abcam | #ab150077 | IF | 1:200 |
| Mouse IgG | Millipore | #12-371 | IP | 1:200 |

CST, Cell Signaling Technology; SAB, Signalway antibody; WB, western blot; IP, immunoprecipitation; IF, immunofluorescence; IHC, immunohistochemistry.

**References:**

1. Manning G, Whyte DB, Martinez R, Hunter T, Sudarsanam S. The protein kinase complement of the human genome. Science. 2002;298: 1912-1934.

2. McGinnis CS, Murrow LM, Gartner ZJ. DoubletFinder: Doublet Detection in Single-Cell RNA Sequencing Data Using Artificial Nearest Neighbors. Cell Syst. 2019;8: 329-337 e324.

3. Hao Y, Hao S, Andersen-Nissen E, et al. Integrated analysis of multimodal single-cell data. Cell. 2021;184: 3573-3587 e3529.

4. Korsunsky I, Millard N, Fan J, et al. Fast, sensitive and accurate integration of single-cell data with Harmony. Nat Methods. 2019;16: 1289-1296.

5. Tirosh I, Izar B, Prakadan SM, et al. Dissecting the multicellular ecosystem of metastatic melanoma by single-cell RNA-seq. Science. 2016;352: 189-196.

6. Qiu X, Mao Q, Tang Y, et al. Reversed graph embedding resolves complex single-cell trajectories. Nat Methods. 2017;14: 979-982.

7. Gulati GS, Sikandar SS, Wesche DJ, et al. Single-cell transcriptional diversity is a hallmark of developmental potential. Science. 2020;367: 405-411.

8. Wu Y, Yang S, Ma J, et al. Spatiotemporal Immune Landscape of Colorectal Cancer Liver Metastasis at Single-Cell Level. Cancer Discov. 2022;12: 134-153.

9. Elosua-Bayes M, Nieto P, Mereu E, Gut I, Heyn H. SPOTlight: seeded NMF regression to deconvolute spatial transcriptomics spots with single-cell transcriptomes. Nucleic Acids Res. 2021;49: e50.

10. Pham D, Tan X, Balderson B, et al. Robust mapping of spatiotemporal trajectories and cell-cell interactions in healthy and diseased tissues. Nat Commun. 2023;14: 7739.

11. Lim SK, Lu SY, Kang SA, et al. Wnt Signaling Promotes Breast Cancer by Blocking ITCH-Mediated Degradation of YAP/TAZ Transcriptional Coactivator WBP2. Cancer Res. 2016;76: 6278-6289.

12. Zhou Y, Tan Z, Xue P, Wang Y, Li X, Guan F. High-throughput, in-depth and estimated absolute quantification of plasma proteome using data-independent acquisition/mass spectrometry ("HIAP-DIA"). Proteomics. 2021;21: e2000264.

13. Xuan Y, Bateman NW, Gallien S, et al. Standardization and harmonization of distributed multi-center proteotype analysis supporting precision medicine studies. Nat Commun. 2020;11: 5248.

14. Zhou Y, Hu L, Tang W, et al. Hepatic NOD2 promotes hepatocarcinogenesis via a RIP2-mediated proinflammatory response and a novel nuclear autophagy-mediated DNA damage mechanism. J Hematol Oncol. 2021;14: 9.

15. Vavricka SR, Musch MW, Chang JE, et al. hPepT1 transports muramyl dipeptide, activating NF-kappaB and stimulating IL-8 secretion in human colonic Caco2/bbe cells. Gastroenterology. 2004;127: 1401-1409.
