# Supplementary material for: RIPK2 promotes colorectal cancer metastasis by protecting YAP degradation from ITCH-mediated ubiquitination
Source: Cell Death Dis. 2025 Apr 4;16(1):248. doi: 10.1038/s41419-025-07599-9 (PMC11971272; doi:10.1038/s41419-025-07599-9)
Supplement: Supplementary file 2 — Supplementary Figures [file 41419_2025_7599_MOESM2_ESM.docx]

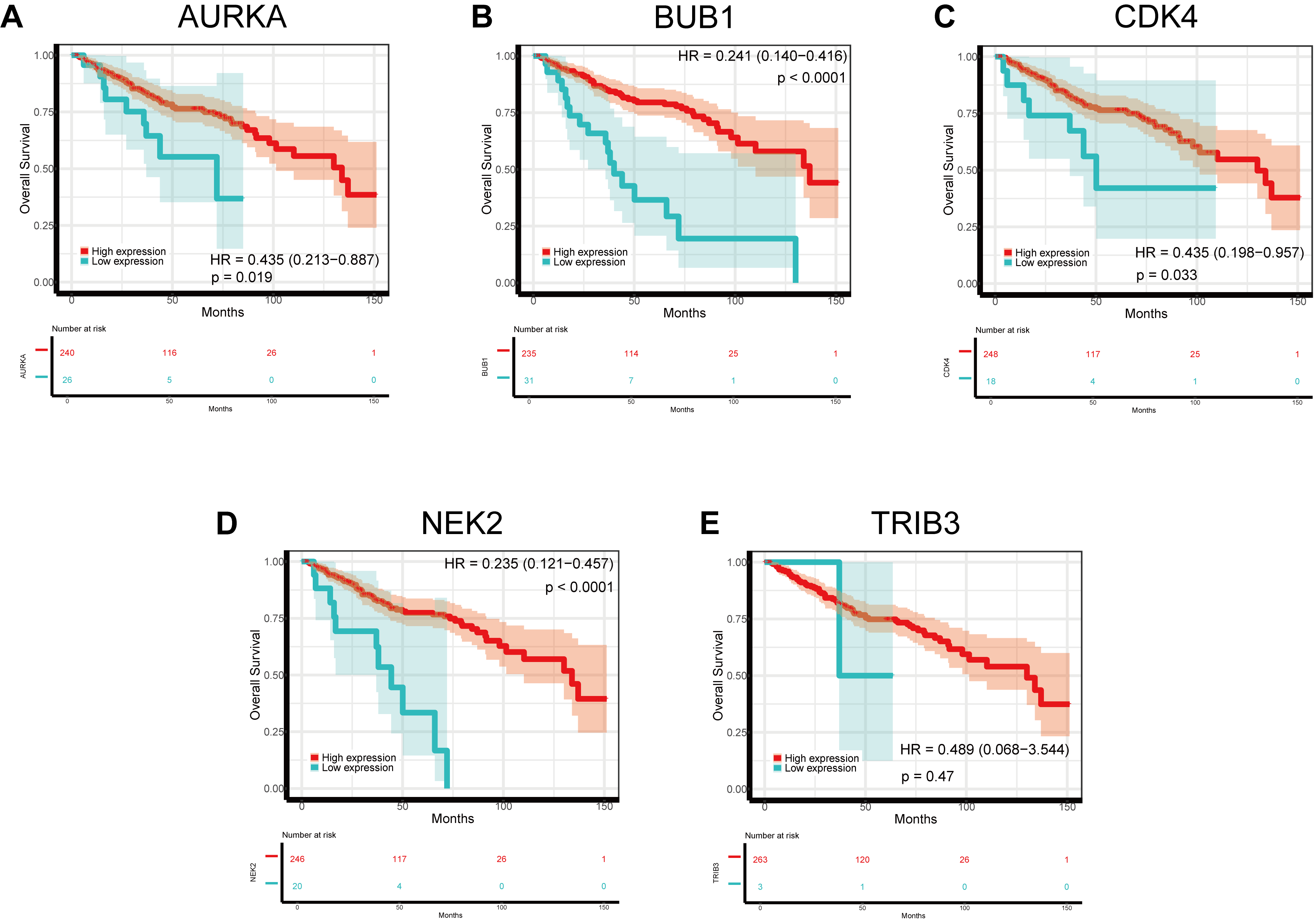


**Figure S1. Kaplan-Meier survival analysis of other five differential expressed kinases in CRC cohort.**

(A-E) Overall survival analysis of AURKA (A), BUB1 (B), CDK4 (C), NEK2 (D), and TRIB3 (E) in CRC cohorts, including GSE39582, GSE41258, GSE87211 and TCGA (COAD and READ), using Kaplan-Meier survival analysis.


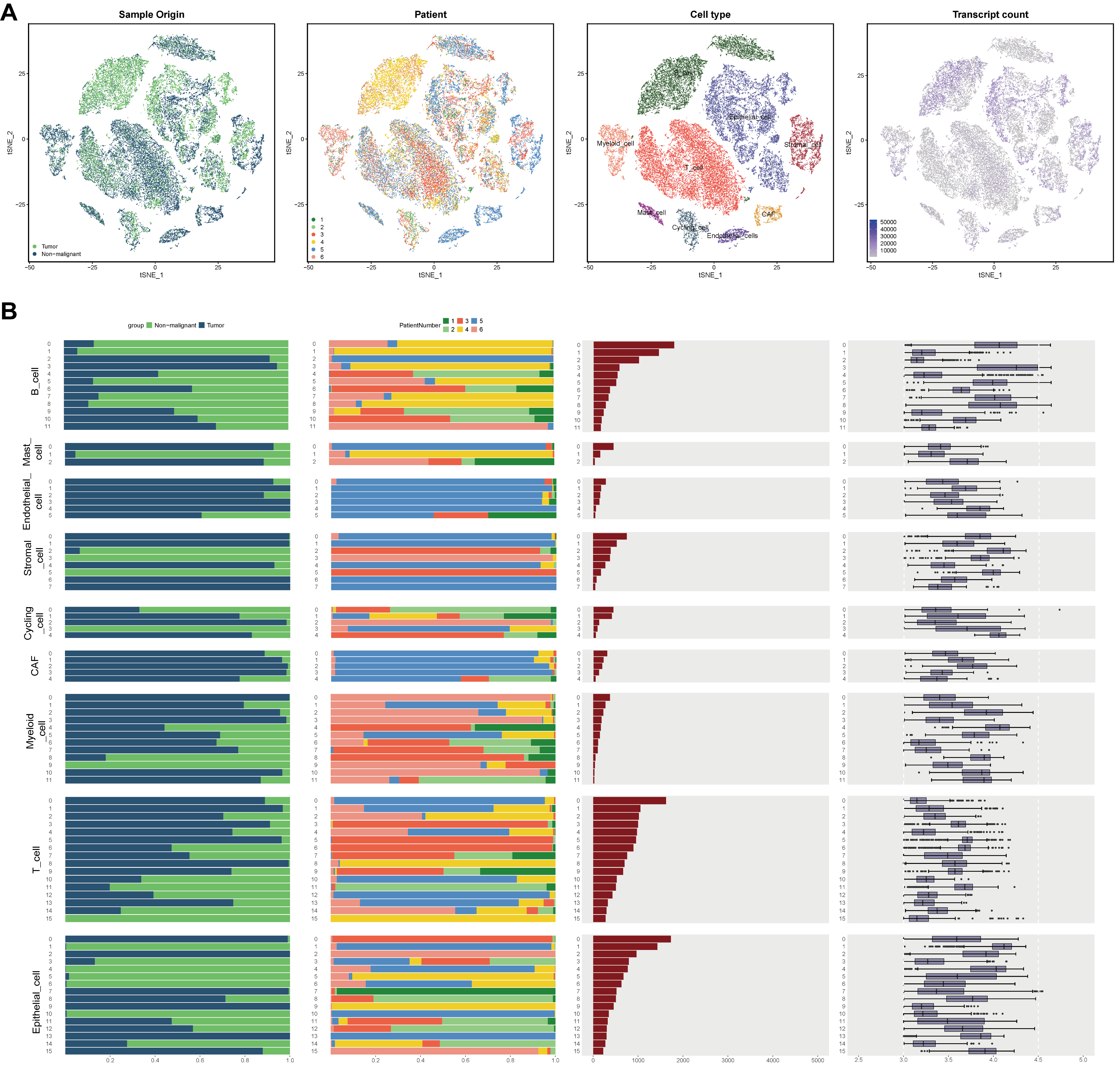


**Figure S2. Overview of 37,277 single cells from CRC primary tumors and non-malignant colon samples.**

(A) t-SNE plot of the 37,277 cells profiled here, with each cell color-coded for (left to right): its sample type of origin (tumor or non-malignant colon), the corresponding patient, the associated cell type, and the number of transcripts (UMIs) detected in that cell (log scale as defined in the inset). (B) For each of the 83 cell subclusters (left to right): the fraction of cells originating from the 6 non-malignant and 6 paired tumor samples, the fraction of cells originating from each of the 6 patients, the number of cells and box plots of the number of transcripts.


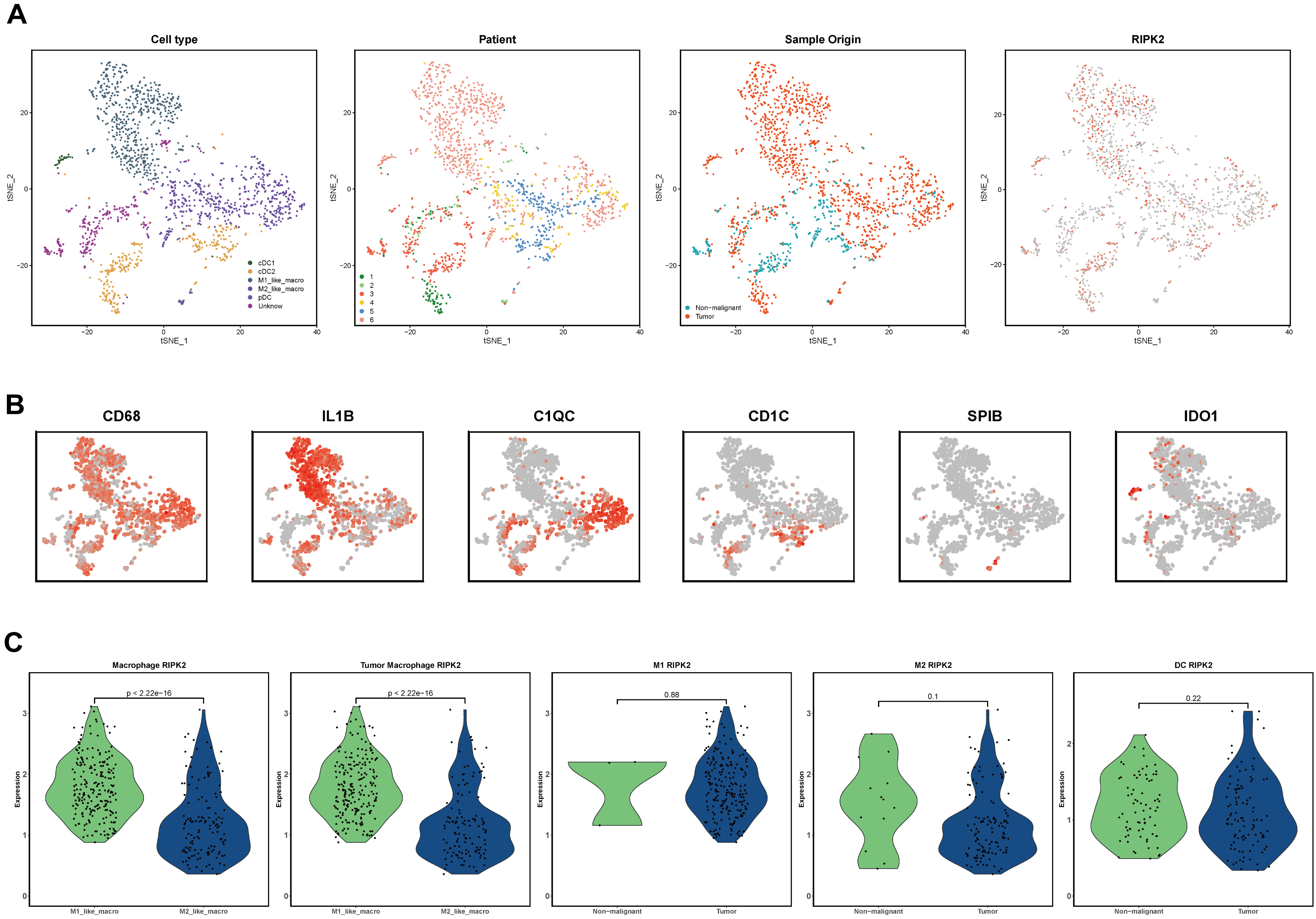


**Figure S3. Analysis of distribution of RIPK2 expression in the Myeloid cell cluster.**

(A) t-SNE plot of the 1,726 cells profiled here, with each cell color-coded for (left to right): its cell type of macrophages and dendritic cells (DCs), the corresponding patient, sample type of origin (tumor or non-malignant colon), RIPK2 counts detected in these cells. (B) Expression of marker genes for the cell types defined above each panel. (C) Differentially RIPK2 expression was detected in macrophages or DCs (left to right): M1-like macrophages and M2-like macrophages from all samples, M1-like macrophages and M2-like macrophages from tumor samples, tumor and non-malignant samples from M1-like macrophages, tumor and non-malignant samples from M2-like macrophages, tumor, and non-malignant samples from DCs.


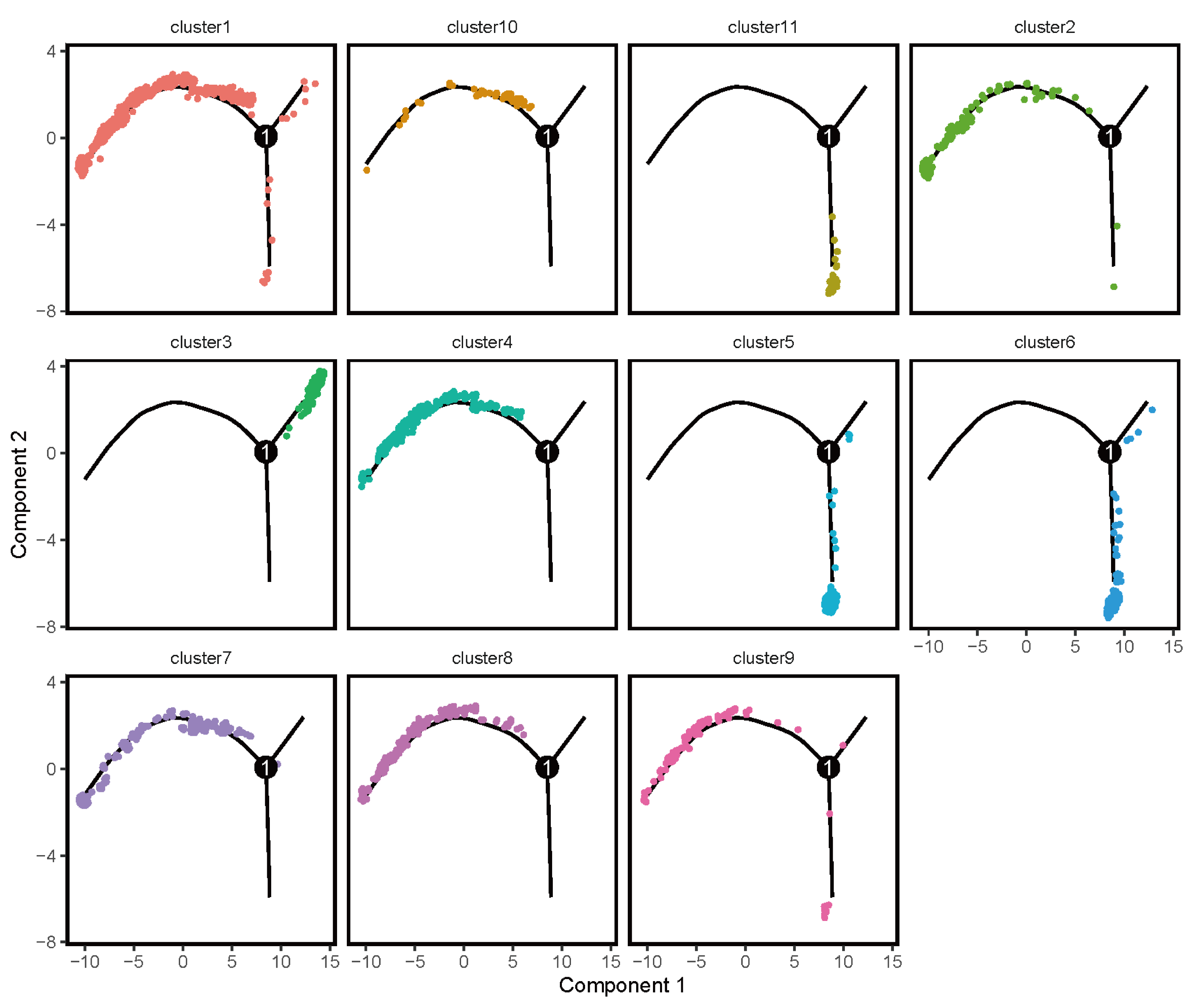


**Figure S4. Pseudotime analysis of each malignant epithelial cell subcluster.**

The trajectory plot illustrates the dynamics of malignant epithelial cell subclusters.


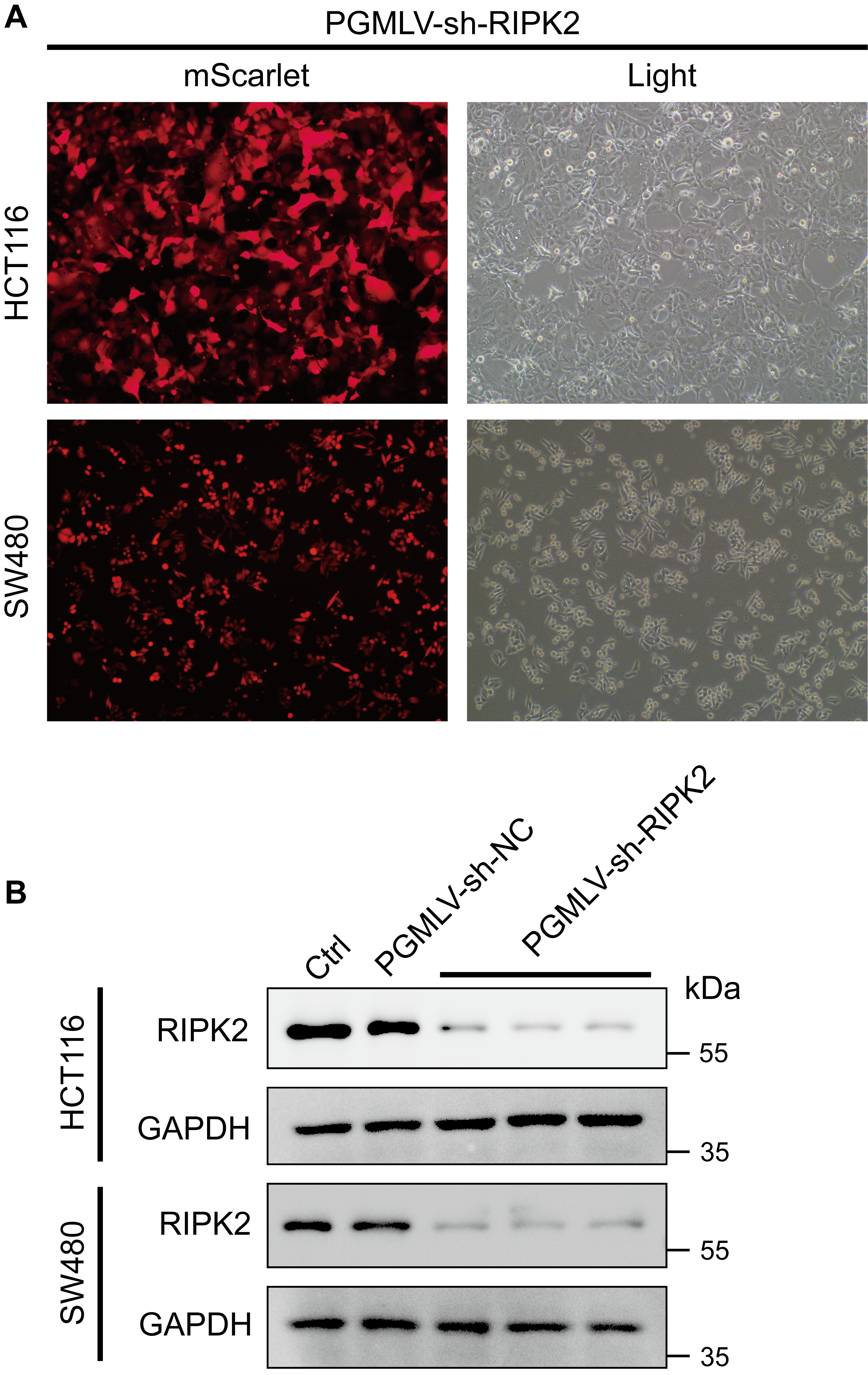


**Figure S5. Efficiency of RIPK2 knockdown with sh-RIPK2 lentivirus.**

(A) Fluorescence transfection efficiency of CRC cells infected with sh-RIPK2 lentivirus. (B) Efficiency of RIPK2 knockdown in CRC cells was detected using western blotting.


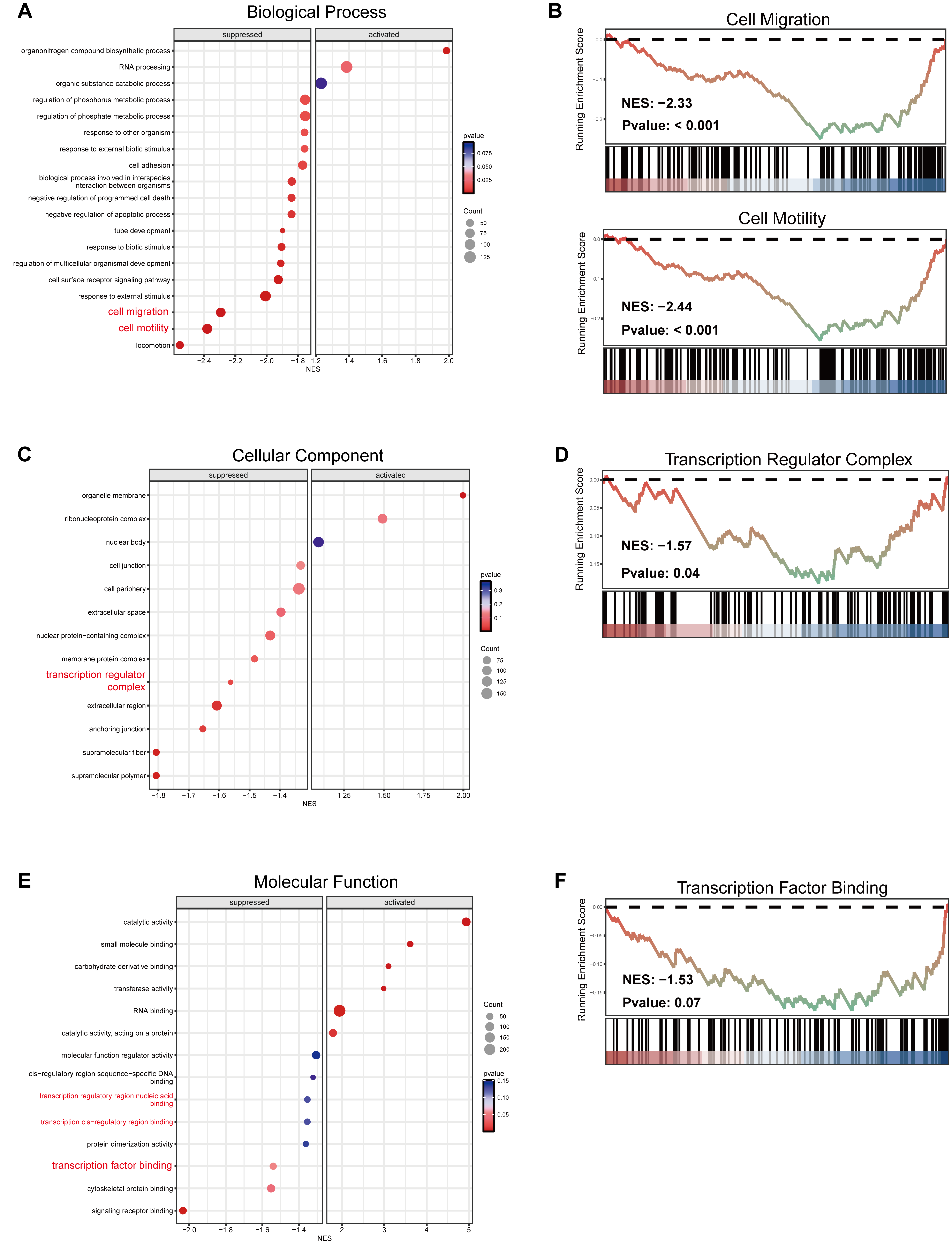


**Figure S6. GO analysis of DEPs from proteomics by GSEA.**

(A, C, E) GO analysis of DEPs by GSEA in BP (A), CC (C), MF (E). (B) In biological process, GSEA plots showing inhibition of cell migration and cell motility in the RIPK2-KD group. (D) In cellular component, GSEA plots showing inhibition of transcription regulator complex in the RIPK2-KD group. (F) In molecular function, GSEA plots showing inhibition of transcription factor binding in the RIPK2-KD group.


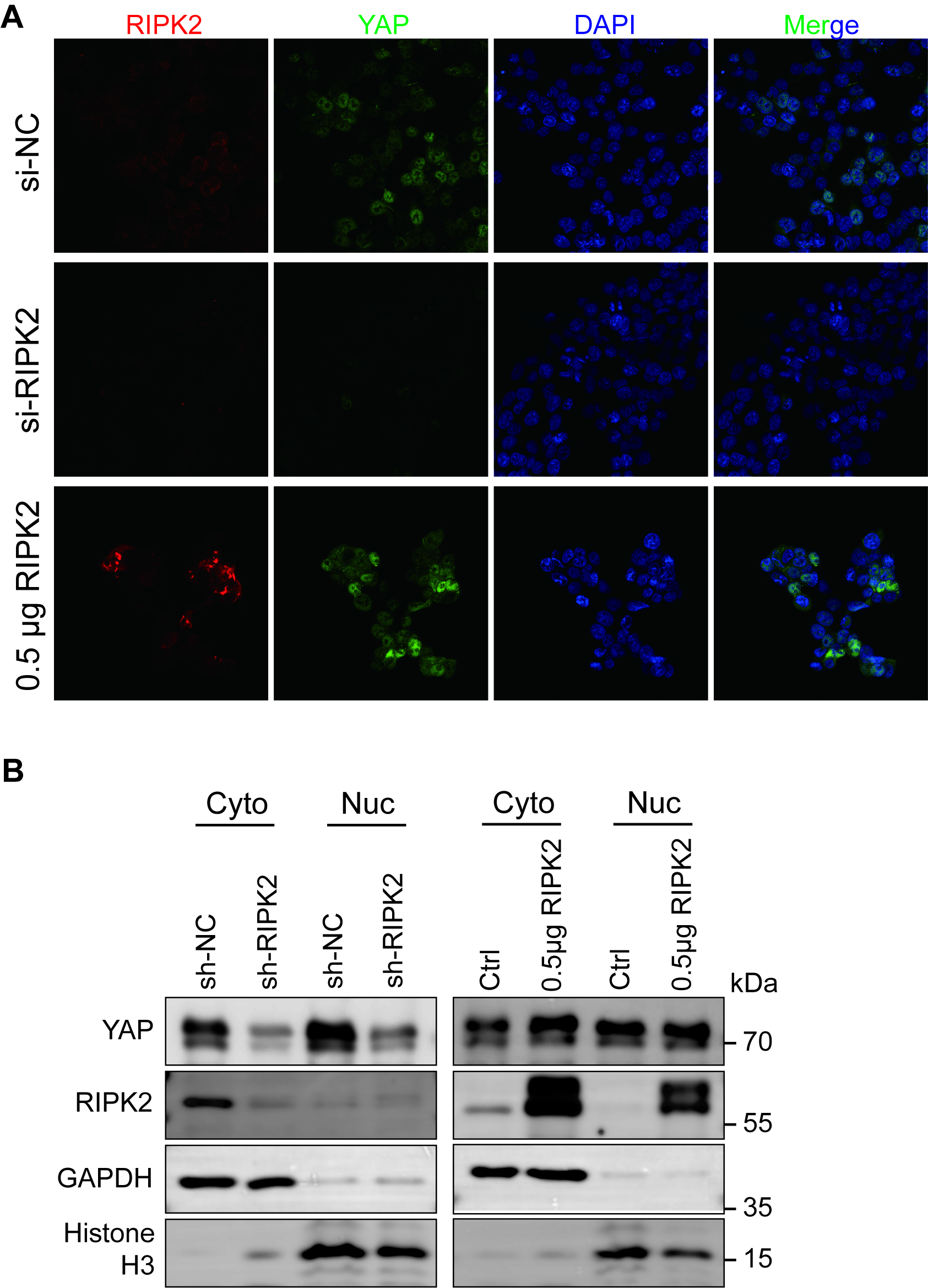


**Figure S7. RIPK2 stabilizes YAP expression but does not promote its nuclear localization.**

(A) IF analysis of whether the distribution of YAP protein in cytoplasm and nucleus is influenced by the expression interference of RIPK2 in HCT116 cells. (B) Immunoblots of the indicated proteins of cytoplasm lysates and nucleus lysates from HCT116 cells transfected with sh-RIPK2 plasmids (left) or RIPK2-expressed plasmids (right), compared with control cells.


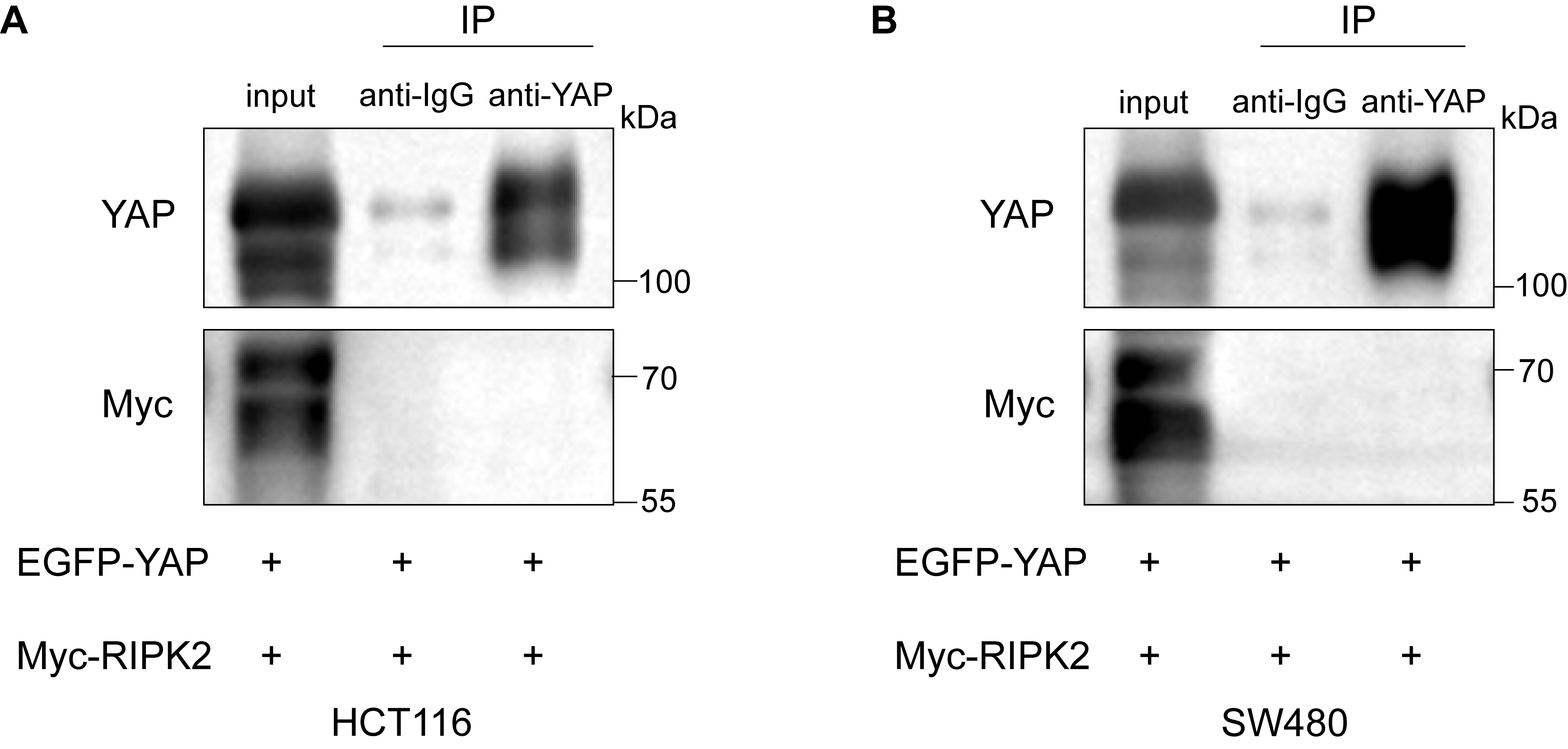


**Figure S8. RIPK2 and YAP do not exhibit a significant direct interaction with each other.**

(A-B) Immunoprecipitation (IP) assays were performed in HCT116 (A) and SW480 (B) cells transiently transfected with Myc-RIPK2 and EGFP-YAP. Lysates were immunoprecipitated with anti-YAP or control IgG antibodies, followed by immunoblotting (IB) with anti-Myc (for RIPK2) and anti-YAP antibodies. Input lanes represent total protein expression.
