# Supplementary material for: RIPK2 promotes colorectal cancer metastasis by protecting YAP degradation from ITCH-mediated ubiquitination
Source: Cell Death Dis. 2025 Apr 4;16(1):248. doi: 10.1038/s41419-025-07599-9 (PMC11971272; doi:10.1038/s41419-025-07599-9)
Supplement: Supplementary file 3 — Additional Supplementary Files [file 41419_2025_7599_MOESM3_ESM.zip › Description of Additional Supplementary Files.docx]

**Description of Additional Supplementary Files**

**Additional Supplementary Files 1**. The clinical information of 152 CRC patients.

**Additional Supplementary Files 2**. Clinical information of five cohorts (GSE39582, GSE41258, GSE87211, COAD and READ).

**Additional Supplementary Files 3**. Detailed information of GSE231559 and GSE245552.

**Additional Supplementary Files 4**. Detailed information on marker genes for various cell types.

**Additional Supplementary Files 5**. DIA proteomics identification and quantification of proteins in negative control and RIPK2 knockdown HCT116 cells.
